# Taxonomic Revision of the Globally Widespread Cryptic *Rhynchomonas* Morphospecies Complex

**DOI:** 10.1111/jeu.70137

**Published:** 2026-11-06

**Authors:** Julian Wagenhofer, Frank Nitsche, Hartmut Arndt

**Affiliations:** ^1^ General Ecology, Institute of Zoology University of Cologne Cologne Germany

**Keywords:** 18S rDNA gene phylogeny, heterotrophic flagellates, kinetoplastids, Rhynchomonadidae, salinity tolerance, synapomorphy

## Abstract

Rhynchomonads are ubiquitously distributed, small, colorless, heterotrophic flagellates with confirmed occurrences in diverse aquatic and soil habitats. To date, *Rhynchomonas nasuta* (Stokes 1888) Klebs 1892 is the commonly accepted species. However, phylogenetic studies revealed considerable genetic divergence, suggesting that isolates of this highly successful morphotype do not all belong to the same species. To resolve this discrepancy, we conducted an integrative taxonomic study combining morphological, autoecological, and molecular phylogenetic methods. While morphological characteristics proved insufficient for reliable species delineation, the integration of salinity tolerance experiments and phylogenetic analyses (18S and 28S rDNA sequences) revealed distinct and well‐supported clades. The obtained results support the description of nine new species (*Rhynchomonas minimamirounga* n. sp., *Rhynchomonas reesi* n. sp., *Rhynchomonas coloniensis* n. sp., *Rhynchomonas eder* n. sp., *Rhynchomonas mylnikovi* n. sp., *Rhynchomonas baltica* n. sp., *Rhynchomonas iris* n. sp., *Rhynchomonas pyschnyi* n. sp., *Rhynchomonas oltome* n. sp.) within the genus *Rhynchomonas*. Additionally, we propose a neotype for *Rhynchomonas nasuta* and confirm *Rhynchomonas metabolita* Pshenin 1964 as valid species.

## 1 Introduction

Heterotrophic nanoflagellates are among the most diverse and abundant eukaryotes globally (Fenchel 1987). They play a crucial role in ecosystems by acting as nutrient remineralizers, shaping bacterial community structure and transferring large amounts of bacterial biomass to higher trophic levels (Jürgens and Matz 2002; Weber et al. 2012). Kinetoplastids form a significant part of the protist community with high abundances in benthic habitats and exhibit great diversity (Arndt et al. 2000; Hohlfeld et al. 2023). Bodonids, as important representatives of the kinetoplastids, are omnipresent in aquatic and soil ecosystems and considered to be of great importance, especially the phagotrophic morphotypes *Neobodo* and *Rhynchomonas* (Flegontova et al. 2018; Mukherjee et al. 2015). Bodonids developed adaptations to survive even harshest conditions like the abyssal deep‐sea, hypersaline salars in the desert or the Antarctic continent (Arndt et al. 2000; Hohlfeld et al. 2021; Rybarski et al. 2021). They also exhibit a variety of feeding strategies that allow different species to thrive in distinct habitats, that is filter, interception or raptorial feeding (Boenigk and Arndt 2002; Fenchel 1987). Latter strategy is used by the kinetoplastids morphospecies *Rhynchomonas nasuta* (Boenigk and Arndt 2000). Originally described as *Heteromita nasuta* Stokes 1888 and renamed to *Rhynchomonas nasuta* (Stokes 1888) Klebs 1892 (order Neobodonida) (Figure 1A), this flagellate morphospecies can be easily identified through its pronounced anterior proboscis, which is about half as long as the cell (Prokina and Philippov 2018) that it uses to search for food particles (Swale 1973). After earlier morphological and behavioral investigations (Bělař 1916; Klebs 1892), Swale (1973) published an extensive study on the morphology, behavior, and occurrence of the *Rhynchomonas* morphotype (Figure 1B). It has two flagella, the shorter anterior being pressed against the proboscis, thereby supporting the movement of the proboscis and making it virtually invisible during light microscopical observations. The posterior flagellum trails behind the cell on the substrate. Both flagella are rooted in the anterior part of the cell. *Rhynchomonas* cells usually creep along the substratum with its proboscis that contains the cytopharynx sweeping up and down until encountering food cells that are consequently engulfed and ingested or rejected (Boenigk and Arndt 2000). The organism is capable of chemosensory responses during the grazing process (Millán et al. 2010). *Rhynchomonas metabolita* Pshenin 1964 was the second species of this genus, isolated from the deep sea of the Mediterranean (Figure 1C; Pshenin 1964). Rhynchomonads are widely known as early biofilm colonizer (Artolozaga et al. 1997; Weitere et al. 2005) and heterotrophic consumer of bacterial cells (Christaki et al. 2005). Bręk‐Laitinen and Ojala (2011) showed that they also can ingest picoalgae (*Choricystis* sp.). Individual cells were reported to measure 4–7 μm in length and 2–3.5 μm in width (Boenigk and Arndt 2000; Klebs 1892; Prokina and Philippov 2018; Stokes 1888; Swale 1973). Boenigk and Arndt (2000) reported a cell volume of 18.5 ± 5.4 μm^3^, Christaki et al. (2005) found differing cell volumes for individuals of the same isolate when either growing on bacteria (14.2 ± 8.7 μm^3^) or the picoeukaryote *Ostreococcus tauri* (9.4 ± 8.7 μm^3^). *Rhynchomonas* was the subject of interest in various experimental studies, that is, for grazing studies (Boenigk and Arndt 2000, 2002; Caron 1987), abiotic tolerance studies (Arndt et al. 2000; Atkins et al. 1998), and studies on defense mechanisms of prokaryotes during protozoan grazing (Sun et al. 2015; Weitere et al. 2005). Morphotypes resembling *Rhynchomonas* or sequences associated with *Rhynchomonas* genotypes were reported from globally very different habitats: marine pelagic and deep sea (Arndt et al. 2003; Atkins et al. 1998; Hohlfeld and Arndt 2022; Scheckenbach et al. 2005; Schoenle et al. 2021; Tikhonenkov et al. 2015) as well as brackish waters (Backe‐Hansen and Throndsen 2002; Von der Heyden et al. 2004; Weber et al. 2017), saline lagoons (Lugo et al. 1998; Patterson and Simpson 1996; Post et al. 1983; Prokina 2020), freshwaters, including rivers, lakes and groundwater (Bancsi and Hamar 1981; Butler 1999; Loquay et al. 2009), and soil (Domonell et al. 2013; Ekelund and Patterson 1997; Foissner 1991; Frederiksen et al. 2001). Furthermore, they have been verified in many different extreme habitats such as geothermal sites (Aguilera et al. 2010), hypersaline (Patterson and Simpson 1996; Prokina 2020), and anoxic (Bernard et al. 2000; Edgcomb et al. 2011) habitats, sea ice brine (Ikävalko and Gradinger 1997), or the gut of different host organisms (Parisi 1910; Votýpka et al. 2021).

**FIGURE 1:**
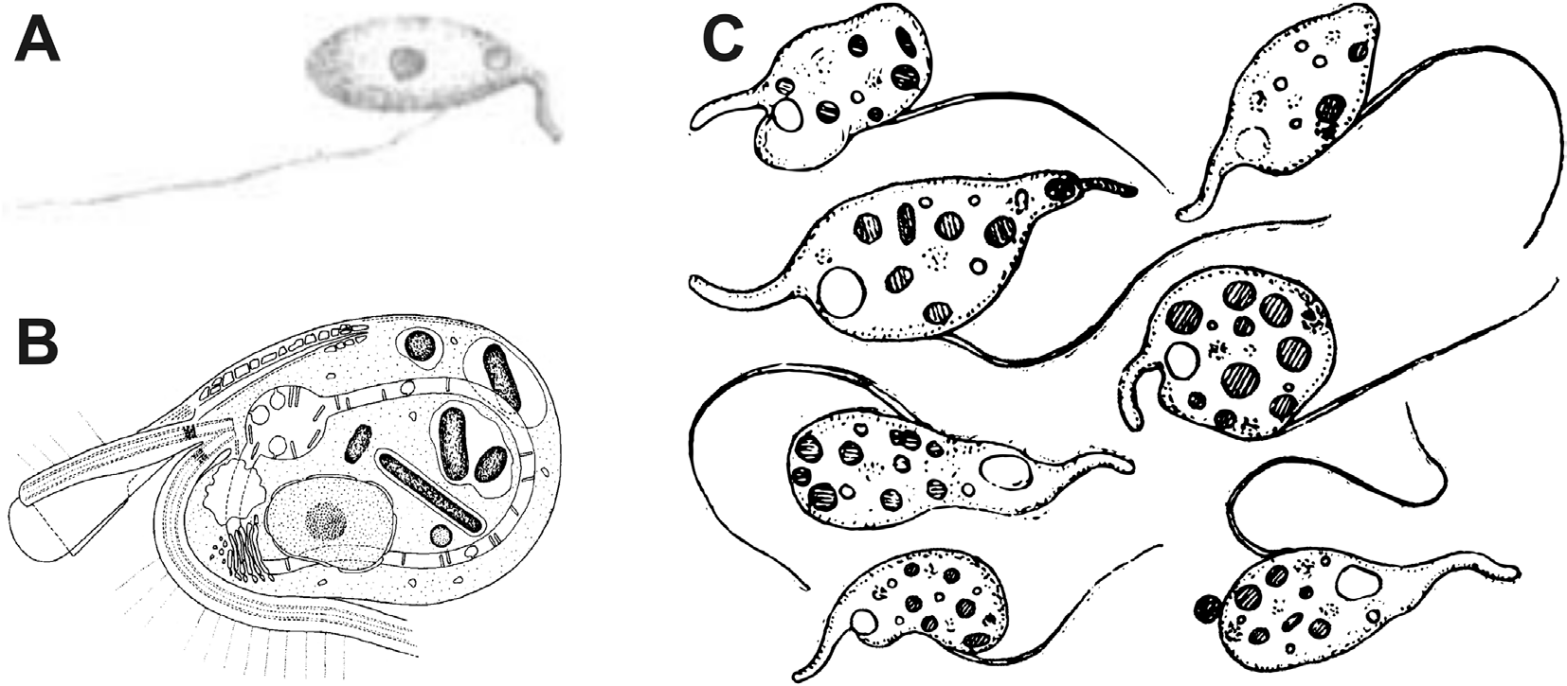
Illustrations of *Rhynchomonas* by different authors, modified after Stokes (1888), Swale (1973), and Pshenin (1964). (A) Drawing of the original description of *Rhynchomonas nasuta* (*Heteromita nasuta*) by Stokes (1888), (B) schematic drawing of *Rhynchomonas* by Swale (1973) according to ultrastructural studies, (C) drawing of the original description of *Rhynchomonas metabolita* by Pshenin (1964).

*Rhynchomonas* isolates from different microhabitats have been shown to exhibit distinct tolerances regarding abiotic parameters, including high hydrostatic pressure levels (Atkins et al. 1998; Živaljić et al. 2018) and salinity (Arndt et al. 2000; Ekelund 2002; Mylnikov 1983). At least some isolates have been shown to be able to survive unfavorable conditions through the formation of cysts (Atkins et al. 1998). Studies on the taxonomy and diversity have historically been based on distinguishing species by morphological characteristics (Patterson and Lee 2000). This changed with the introduction of molecular techniques, which has since become a crucial tool for the identification of different genotypes and species. For estimating the diversity of protists, the discussion on the ubiquitous dispersal hypothesis versus the moderate endemicity model has been of major importance (Foissner 2006), with the latter being nowadays accepted to more likely represent the global species richness suggesting the existence of cosmopolitan as well as endemic species (Bass et al. 2007; Van der Gast 2015). *Rhynchomonas‐*like cells have been observed globally, yet molecular studies show the existence of a high degree of genetic variation within this morphotype, making it an ecologically very successful cryptic species complex. The cryptic character of the *Rhynchomonas* species complex has been the subject of previous studies with repeated calls for phylogenetic and taxonomic revision (Birky et al. 2010; Callahan et al. 2002; Domonell et al. 2013; Scheckenbach et al. 2005; Von der Heyden et al. 2004). The goal of this study was therefore to start to resolve this cryptic *Rhynchomonas* morphotype complex and uncover its hidden diversity by conducting an integrative study on the morphology, autoecology, biogeography, and phylogeny of different *Rhynchomonas* genotypes isolated from various different regions of the world.

## 2 Materials and Methods

### 2.1 Sampling, Isolation, and Cultivation

Samples were taken from diverse marine, brackish, and freshwater environments and include benthic as well as pelagic habitats. All isolates within the framework of this study were obtained between 1995 and 2023 (Figure 2, Table 1). Monoclonal cultures were obtained by picking single cells from diluted samples using a micromanipulator (Patchman MP2; Eppendorf, Hamburg, Germany) or applying the liquid aliquot method (Butler and Rogerson 1995). The monoclonal cultures were inoculated in 50 mL tissue culture flasks (Falcon, Durham, USA) filled with 35, 23, or 8 PSU (Practical Salinity Units in ‰) Schmaltz‐Pratt (SP) medium (Goryatcheva 1971) (SP, 35 PSU [L^−l^]: 28.15 g NaCl, 0.67 g KCl, 5.51 g MgCl_2_ × 6H_2_O, 6.92 g MgSO_4_ × 7H_2_O, 1.45 g CaCl_2_ × 2H_2_O, 0.1 g KNO_3_, 0.01 g K_2_HPO_4_ × 3H_2_O), artificial seawater (ASW) medium prepared from reef salt (Aqua Medic, Bissendorf, Germany) (ASW, 35 PSU [L^−l^]: 11 g Na, 1.2 g Mg, 0.42 g Ca, 0.35 g K, 19.7 g Cl, 2.2 g SO_4_, 0.18 g HCO_3_, 0.016 g Sr) or Wright Chu (WC) medium (Guillard and Lorenzen 1972) (WC [L^−l^]: 42.5 g NaNO_3_, 36.97 g MgSO_4_ × 7H_2_O, 36.76 g CaCl_2_ × 2H_2_O, 4.35 g K_2_HPO_4_, 31.5 g NaHCO_3_, 21.2 g Na_2_O_3_Si × 5H_2_O, 3.15 g FeCl_3_ × 6H_2_O, 4.36 g Na_2_EDTA, 0.01 g CuSO_4_ × H_2_O, 0.022 g ZnSO_4_ × 7H_2_O, 0.01 g CoCl_2_ × 6H_2_O, 0.18 g MnCl_2_ × 4H_2_O, 0.006 g NaMoO_4_ × 2H_2_O, 0.13 g H_3_BO_3_) depending on each isolate's origin. A sterile filtered vitamin solution (0.1 g/L Thiamine HCl, 0.5 mg/L Biotin, 0.5 mg/L Cyanocobalamin) was added to the WC medium after autoclavation. Two quinoa grains were added to each culture as a carbon source for the associated bacterial communities. For long‐term storage and cultivation, the cultures were transferred to 23 and 35 PSU ASW medium or WC medium and stored in climate chambers (10°C and 20°C) or stored away deep‐frozen in Liquid N_2_ (Simon 1972).

**FIGURE 2:**
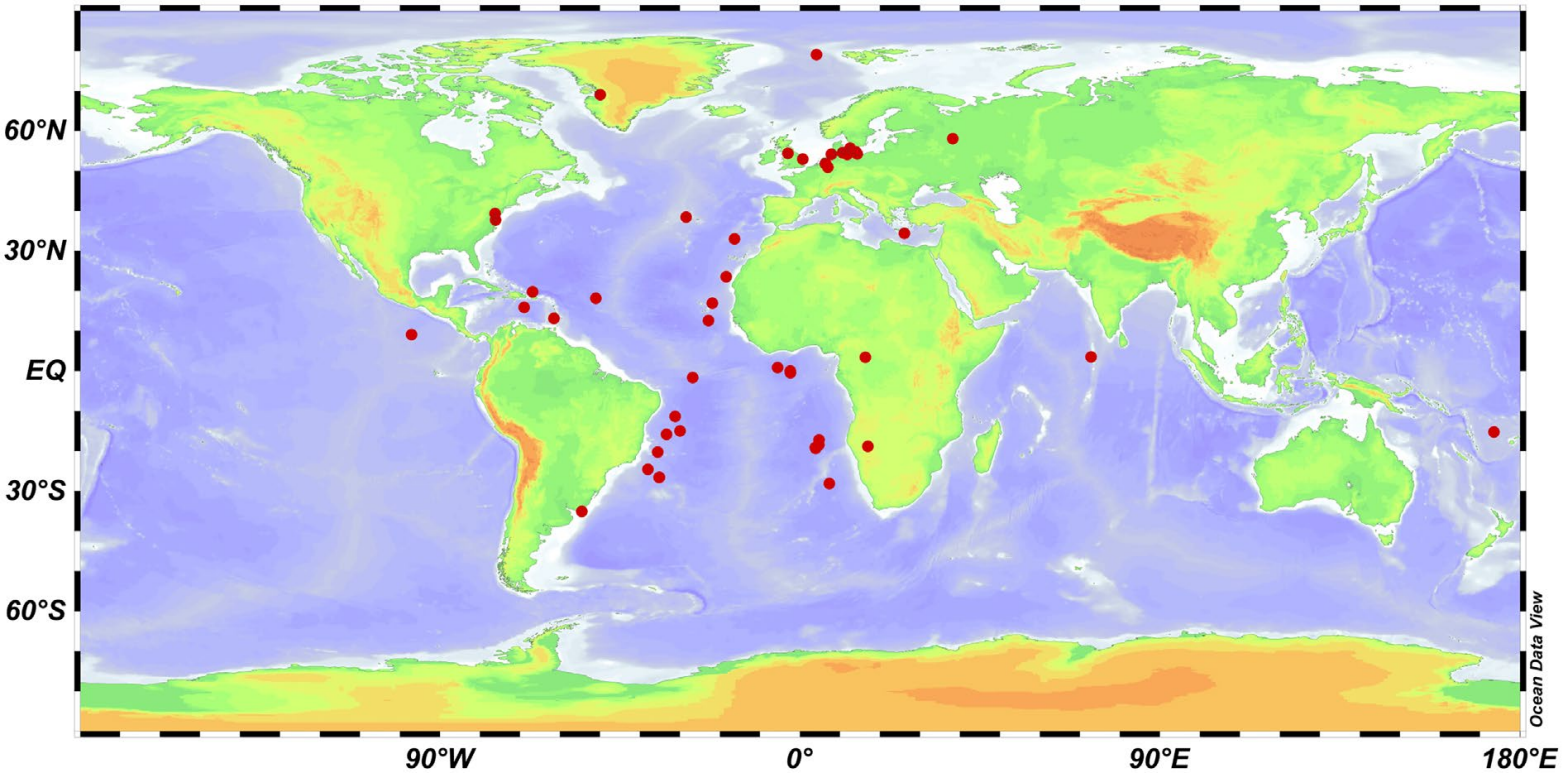
Origin of rhynchomonad isolates used for phylogenetic analyses within this study. For three isolates (HFCC1504, AY425022, AY425023), no exact data on origin could be obtained. Image was generated with Ocean Data View. See also Figure S5 and the records of GBIF Secretariat (2023).

**TABLE 1:** Isolates (*n* = 90) used for phylogenetic analyses within this study.

Isolate	Geographical origin	Coordinates	Habitat	Depth (m)
HFCC17	Russia, Borok Yaroslavl, Dystrophic Pond	58°3′42.99984 N, 38°14′4.99992 E	fw	n/a
HFCC18	SAO, Angola Basin	−19°6′54 N, 3°52′0.0012 E	m, Water column	1
HFCC99	NAO	79°4′15.6 N, 4°9′7.2 E	m, Sediment	2484
**HFCC111**	Eastern Mediterranean, Pliny trench	34°25′4.98 N, 26°7′6.1788 E	m, Hard substrate	4390
**HFCC147**	Deep Sea, SAO	−14°58′43.2012 N, −29°57′28.1988 E	m, Sow	5127
**HFCC148**	SAO	−26°32′17.9988 N, −35°6′32.4 E	m	n/a
**HFCC149**	SAO	−26°34′3.6012 N, −35°13′11.3988 E	m, Sow	4494
**HFCC156**	Azores, Faial, at foot of Mont'Ana Seamount	38°28′24.9996 N, −28°27′15.9984 E	m, Sediment	450
HFCC171	Deep Sea, Puerto Rico Trench	19°46′0.5988 N, −66°49′59.4012 E	m, Sow	8346
HFCC173	Deep Sea, Puerto Rico Trench	19°46′0.5988 N, −66°49′59.4012 E	m, Sargassum	n/a
HFCC196	Deep Sea, Puerto Rico Trench	19°46′1.2 N, −66°49′0.0012 E	m, Sediment	8337
**HFCC231**	Namibia, Fisher's Pan Etosha National Park	−18°47′35.0016 N, 16°59′25.0008 E	Salt pan, sediment, 7 PSU	n/a
**HFCC234**	Germany, Cologne, River Rhine	50°54′25.0776 N, 6°58′42.384 E	fw, Surface water	0
HFCC302	EAO, Angola Basin	−19°19′48 N, 3°55′36.0012 E	m, Sediment	5425
HFCC303	EAO, Angola Basin	−18°19′30 N, 4°43′0.0012 E	m, Sediment	5414
HFCC315	EAO, Angola Basin	−18°25′18.0012 N, 4°43′59.9988 E	m, Sediment	5392
HFCC316	EAO, Angola Basin	−17°11′35.9988 N, 4°45′54 E	m, Sediment	5415
HFCC319	Germany, Cologne, River Rhine	50°54′25.0776 N, 6°58′42.384 E	fw, Surface water	0
HFCC322	SAO, Angola Basin	−19°6′54 N, 3°52′0.0012 E	m, Sediment	5423
HFCC332	AO (Equator), Eastern Guinea Abyssal Plain	0°0′0 N, −2°25′0.0012 E	m, Sediment	5063
HFCC333	NAO, Western Guinea Abyssal Plain	0°49′59.9988 N, −5°34′59.9988 E	m, Sediment	5140
HFCC334	SAO, Eastern Guinea Abyssal Plain	0°0′0 N, −2°25′0.0012 E	m, Sediment	5063
HFCC335	SAO, Northern Cape Abyssal Plain	−28°6′42.0012 N, 7°20′48.0012 E	m, Sediment	5038
HFCC337	SAO, Western Guinea Abyssal Plain	0°49′59.9988 N, −5°34′59.9988 E	m, Sediment	5139
HFCC338	SAO, Eastern Guinea Abyssal Plain	0°0′0 N, −2°25′0.0012 E	m, Sediment	5063
HFCC339	SAO, Eastern Guinea Abyssal Plain	0°0′0 N, −2°25′0.0012 E	m, Sediment	5063
HFCC340	AO (Equator), Eastern Guinea Abyssal Plain	−0°0′6.0012 N, −2°24′54 E	m, Sediment	5063
HFCC341	AO (Equator), Eastern Guinea Abyssal Plain	−0°0′6.0012 N, −2°24′54 E	m, Sediment	5063
HFCC342	AO (Equator), Eastern Guinea Abyssal Plain	−0°0′6.0012 N, −2°24′54 E	m, Sediment	5063
HFCC343	AO (Equator), Eastern Guinea Abyssal Plain	−0°0′6.0012 N, −2°24′54 E	m, Sediment	5063
HFCC344	SAO, Eastern Guinea Abyssal Plain	0°0′0 N, −2°25′5.9988 E	m, Sediment	5063
HFCC345	AO (Equator), Eastern Guinea Abyssal Plain	0°0′0 N, −2°25′0.0012 E	m, Sediment	5063
HFCC346	SAO, Eastern Guinea Abyssal Plain	−0°0′6.0012 N, −2°24′54 E	m, sediment	5062
HFCC347	AO (Equator), Eastern Guinea Abyssal Plain	0°0′0 N, −2°25′5.9988 E	m, Sediment	5063
HFCC348	AO (Equator), Eastern Guinea Abyssal Plain	0°0′0 N, −2°25′5.9988 E	m, Sediment	5063
HFCC368	SAO, Western Guinea Abyssal Plain	0°49′59.9988 N, −5°35′6 E	m, Sediment	5140
HFCC369	SAO, Western Guinea Abyssal Plain	0°50′6 N, −5°35′6 E	m, Sediment	5136
HFCC370	SAO, Cape Abyssal Plain	−28°6′47.9988 N, 7°20′48.0012 E	m, Sediment	5063
HFCC371	SAO, Guinea Abyssal Plain	0°0′6.0012 N, −2°25′0.0012 E	m, Sediment	5063
HFCC372	NAO, Western Guinea Abyssal Plain	0°49′59.9988 N, −5°35′6 E	m, Sediment	5141
HFCC373	SAO, Guinea Abyssal Plain	0°0′0 N, −2°25′0.0012 E	m, Sediment	5064
HFCC374	NAO, Western Guinea Abyssal Plain	0°49′59.9988 N, −5°34′59.9988 E	m, Sediment	5138
HFCC656	Maldives, coral reef	3°29′23.2008 N, 72°47′59.8992 E	m, Sediment	12
**HFCC752**	Caribbean Sea	15°53′12.9588 N, −68°55′44.4 E	m, Sow	4034
**HFCC826**	Greenland, Ilulissat, freshwater lake	69°3′57.4992 N, −49°53′37.8564 E	fw, Surface water	0
**HFCC830**	South Pacific, Oceania	−15°13′2.1 N, 173°31′12.5004 E	m, Water column	2772
**HFCC1139**	NAO	18°10′0.06 N, −51°0′3.9 E	m, Water column	2500
**HFCC1143**	Caribbean Sea	13°7′39.9 N, −61°29′28.74 E	m, Water column	61
**HFCC1300**	SAO, Montevideo coastline	−35°4′39.4212 N, −54°29′58.92 E	m, Surface water	0
**HFCC1303**	SAO	−24°32′24.54 N, −37°59′12.1812 E	m, Surface water	0
**HFCC1307**	SAO	−15°47′42.18 N, −33°19′22.5012 E	m, Surface water	0
**HFCC1320**	SAO	−11°20′37.9212 N, −31°10′57.6588 E	m, Surface water	0
**HFCC1323**	SAO	−1°39′56.0988 N, −26°47′42.36 E	m, Surface water	0
**HFCC1331**	NAO	16°57′31.3812 N, −21°53′52.7388 E	m, Surface water	0
**HFCC1332**	NAO	12°36′28.62 N, −22°51′9.1188 E	m, Surface water	0
**HFCC1346**	SAO	−20°14′39.7788 N, −35°33′1.1412 E	m, Surface water	0
**HFCC1347**	NAO	23°33′13.32 N, −18°24′18.2988 E	m, Surface water	0
**HFCC1357**	NAO	12°36′28.62 N, −22°51′9.1188 E	m, Surface water	0
**HFCC1420**	Baltic Sea, Fehmarnbelt	54°32′30.0012 N, 10°41′9.6 E	b, Sediment (15–20 cm), 19 PSU	23
**HFCC1421**	Baltic Sea, Fehmarnbelt	54°32′30.0012 N, 10°41′9.6 E	b, Sediment (10–15 cm), 19 PSU	23
**HFCC1435**	Baltic Sea, Oderbank	54°15′0.1332 N, 14°20′57.3468 E	b, Sediment (6–7 cm), 8 PSU	15
**HFCC1504**	Shrimp recirculating aquaculture system using Atlantic seawater	n/a	m	n/a
**HFCC1570**	Madeira, Porto Santo	33°0′22 N, −16°20′50 E	m, Sediment (0–1 cm)	997
**HFCC1630**	Baltic Sea, Fehmarnbelt	54°33′22.2012 N, 10°45′31.2012 E	b, Sediment (1–2 cm), 19 PSU	24
**HFCC1639**	Baltic Sea, Fehmarnbelt	54°32′30.5988 N, 10°41′42.6012 E	b, Sediment (10–15 cm), 19 PSU	23
**HFCC1640**	Baltic Sea, Fehmarnbelt	54°32′30.0012 N, 10°41′9.6 E	b, Sediment (1–2 cm), 19 PSU	23
**HFCC1641**	Baltic Sea, Fehmarnbelt	54°32′30.5988 N, 10°41′42.6012 E	b, Sediment (15–20 cm), 19 PSU	23
**HFCC1642**	Baltic Sea, Fehmarnbelt	54°32′46.2012 N, 10°46′36.5988 E	b, Sediment (15–20 cm), 19 PSU	23
**HFCC1657**	Baltic Sea, Roennebank	54°46′23.394 N, 14°0′55.6992 E	b, Sediment (0–1 cm), 12 PSU	38
**HFCC1658**	North Sea, Helgoland harbor	54°11′0.3012 N, 7°53′22.7004 E	m, Sediment	3
**HFCC1659**	North Sea, Helgoland harbor	54°11′0.3012 N, 7°53′22.7004 E	m, Sediment	3
**HFCC1660**	Baltic Sea, Roennebank	54°46′23.6712 N, 14°0′55.9008 E	b, Sediment (3–4 cm), 12 PSU	38
**HFCC2052**	Germany, Bienener Altrhein	51°48′21.1 N, 6°21′45.4 E	fw, Surface water	0
**HFCC2058**	Germany, Bienener Altrhein	51°48′21.1 N, 6°21′45.4 E	fw, Surface water	0
**HFCC2070**	Germany, Reeser Ward	51°45′18.7 N, 6°27′32.6 E	fw, Sediment (0–1 cm)	n/a
**HFCC2100**	Germany, Cologne, River Rhine	50°54′25.218 N, 6°58′42.1392 E	fw, Benthos cube	0
**HFCC2101**	Germany, Cologne, River Rhine	50°54′25.218 N, 6°58′42.1392 E	fw, Benthos cube	0
**HFCC2102**	Germany, Cologne, River Rhine	50°54′25.218 N, 6°58′42.1392 E	fw, Benthos cube	0
HFCC3310	SAO, Eastern Guinea Abyssal Plain	0°0′0 N, −2°25′0.0012 E	m, Sediment	5064
HFCC3360	SAO, Eastern Guinea Abyssal	−0°26′48.0012 N, −2°20′30.0012 E	m, Sediment	5058
AF174377	USA, Chesapeake Bay	n/a	b, Water column	1
AF174378	East Pacific Ocean, Biovent Serpulid Zone	n/a	m, Hard substrate (basaltic)	2500
AY425023	Sargasso Sea	n/a	m, Water column	35
AY425024	USA, Upper Chesapeake Bay	n/a	b	n/a
AY425025	UK, Burnham Overy Staithe, Norfolk	n/a	b, Sediment	n/a
AY490214	UK, Priest Pot Cumbria	n/a	fw, Algal scrape	n/a
AY998642	Denmark, Copenhagen	n/a	s, Garden soil	n/a
KX420740	Baltic Sea, Heiligendamm	n/a	b, Surface water	0
MW694339	Central African Republic, Dzanga‐Sangha Protected Area	n/a	Host gut (tsetse fly)	n/a
AY425022	No information available	n/a	n/a	n/a

*Note:* New isolates (*n* = 42) are marked in bold.

Abbreviations: AO = Atlantic Ocean, b = brackish, fw = freshwater (0 PSU), m = marine (35–37 PSU), NAO = North Atlantic Ocean, s = soil, SAO = South Atlantic Ocean, sow = sediment overlying water.

### 2.2 DNA Extraction and Sequencing

For DNA extraction from cultures, monoclonal cell cultures were transferred to 50 mL tubes (Sarstedt, Nümbrecht, Germany) and centrifuged (4000 *g*, 20 min, 4°C, Megafuge 2.0 R; Heraeus Instruments). Consequently, the genomic DNA was extracted using the Quick‐gDNA Mini Prep Kit (Zymo Research Europe, Freiburg, Germany), following the manufacturer protocol. For the amplification of the small (18S) and large (28S) subunit ribosomal DNA (rDNA), several primers were used for the PCR, many being designed specifically designed for the sequencing of kinetoplastids. For the 18S SSU, the forward primers 18Sfor‐Bodo (5′‐CTGGTTGATTCTGCCAGTAGT‐3′), Kin740F (5′‐TGTTAAAGGGTTCGTAGTTG‐3′), Kin170‐For (5′‐TGGTCGGTGGAGTGATTTG‐3′) and the reverse primers 18Srev‐Bodo (5′‐TGATCCAGCTGCAGGTTCACC‐3′), Kin740R (5′‐TCAACTACGAACCCTTTAAC‐3′), Kin1720R (5′‐AACAAATCACTCCACCGAC‐3′) (Hillis and Dixon 1991) were used. For the 28S LSU, the forward primer NLF184/21 (5′‐ACCCGCTGAAYTTAAGCATAT‐3′) was combined with the reverse primer NLR1126/22 (5′‐GCTATCCTGAGGGAAACTTCGG‐3′) (Van der Auwera et al. 1994). A final primer concentration of 0.1 μM and a PCR Mastermix (VWR Life Science, Red Taq DNA Polymerase, Hassrode, Belgium) was used. For the 28S LSU primer set NLF184/21 and NLR1126/22, the following steps were conducted: initial denaturation step at 98°C for 2 min, 35 cycles at 98°C for 30 s, 55°C for 30 s, 52°C for 30 s, 72°C for 2 min, and a final elongation step for 10 min at 72°C. For the primer pair 18Sfor‐Bodo and 18Srev‐Bodo, a special PCR with 22 steps was applied: (1) initial denaturation step at 98°C for 2 min, (2) 98°C for 30 s, (3) 60°C for 30 s, (4) 57°C for 30 s, (5) 72°C for 2 min, (6) 98°C for 30 s, (7) 59°C for 30 s, (8) 56°C for 30 s, (9) 72°C for 2 min, (10) 98°C for 30 s, (11) 58°C for 30 s, (12) 54°C for 30 s, (13) 72°C for 2 min, (14) 98°C for 30 s, (15) 57°C for 30 s, (16) 53°C for 30 s, (17) 72°C for 2 min, (18) 98°C for 30 s, (19) 56°C for 30 s, (20) 52°C for 30 s, (21) 72°C for 2 min (Steps 18–21 are repeated for 27 cycles), and (22) a final elongation step for 8 min at 72°C. The length of the resulting PCR products was checked on a 2% agarose gel. Subsequently, it was purified with the FastGene Gel/PCR Extraction Kit (NIPPON Genetics Europe, Düren, Germany) and the obtained products were sequenced at GATC Biotech, Germany.

### 2.3 Phylogeny

All obtained forward and reverse sequences were assembled in BioEdit V7.7.1 (Hall 1999). Eighty‐nine 18S rDNA and 34 28S rDNA *Rhynchomonas* sequences together with *Cryptaulax* sp. ATCC 50746 and two *Neobodo* sp. sequences (HFCC828, HFCC1432) as outgroup (total: 92 sequences) from the Heterotrophic Flagellate Culture Collection Cologne (HFCC) and NCBI with a minimum 18S sequence length of 1000 nucleotides were used and aligned into a concatenated dataset using the ClustalW Multiple Alignment (Thompson et al. 1994) tool in BioEdit. Manual corrections of the concatenated alignment were performed in BioEdit. For the 18S rDNA phylogeny, the alignment was shortened to 3005 nucleotides. Furthermore, the 18S alignment was trimmed using Gblocks (Version 0.91b, at NGphylogeny.fr) (Castresana 2000; Dereeper et al. 2008), which resulted in a trimmed alignment with 1803 positions. The maximum likelihood (ML) analysis was carried out in MEGA V10.2.6 (Felsenstein 1981; Kumar et al. 2018; Nei and Kumar 2000), using the GTR + Γ + I model (partial deletion, 95% site coverage cutoff) with 1000 bootstrap replications. ML analyses were performed for all three alignments. Bayesian inference (BI) analysis was performed on CIPRES Science Gateway V3.3 (Miller et al. 2010) using the MrBayes on XSEDE (3.2.7a) (Ronquist et al. 2012) (nruns = 2, nchains = 4, samplefreq = 100, mcmcdiagn = Yes, diagnfreq = 5000, burninfrac = 0.25) with MCMC generations being set to max. 10,000,000 and a stop value of 0.01 for the average standard deviation of split frequencies, which was reached after 2,710,000 generations. Pairwise genetic distances (p‐distances) for the 18S were calculated in BioEdit using the pairwise alignment function. The long intron (of about 500 nucleotides length), that is present in several isolates, has been removed for p‐distance calculations. Phylogenetic trees were graphically prepared in Inkscape (1.2.2).

### 2.4 Light Microscopy and Morphological Characterization

High‐resolution video‐enhanced differential interference contrast microscopy was performed for the isolates HFCC 148, 1420, 111, 149, 18, 2058, 147, 1570, 2052, 2102, 2101, 1139, 17, 1657, 1435, 99. At least 25 cells were recorded for each isolate. The cultures were observed in a petri dish with a glass cover slip at the bottom with an inverted light microscope (Zeiss Axio Observer A1; Zeiss, Germany) with a water immersion condenser and a 100×/1.4 NA oil immersion objective with differential interference contrast (DIC). Cell cultures were investigated at a 1600× magnification. Videos were recorded with a Zeiss Axiocam 305 mono camera. The program ZEN blue (version 3.5.093.00001) was used for recording, the selection of single frames and the measurement of morphological characteristics (cell length, cell width, length posterior flagellum, length nucleus, width nucleus; exact values in Table S1). Cell length and width were measured from photographs taken during the forward movement of individual cells to ensure comparability. The cell surface area, nucleus surface area, and ratio of cell area/length of the posterior flagellum were calculated consequently.

### 2.5 Scanning Electron Microscopy

For scanning electron microscopy (SEM), fixed monoclonal cultures were prepared on polycarbonate filters (2.0 μm pore size, 25 mm diameter; Nuclepore Track‐Etched Membrane, Cytiva, USA). Fixation was done with cacodylate‐buffered glutaraldehyde (2.5% final concentration) at 4°C for a minimum of 30 min. Fixed samples were consequently dehydrated in an increasing ethanol series (30%, 50%, 70%, 80%, 90%, 96%). Samples were washed twice with each concentration and remained subsequently in each concentration step for 10 min. Filters were never allowed to dry up during the process. After the ethanol series, hexamethyldisilazane (HMDS)–ethanol (1:1) was applied for 15 min (no washing step afterwards), followed by pure HMDS for 2 min. Afterwards, the filters were left to dry. Samples were put onto a sample holder and sputter coated with a 120 Å gold layer. SEM high‐resolution micrographs were taken with a Zeiss Sigma 300‐VP scanning electron microscope (Zeiss, Germany).

### 2.6 Salinity Tolerance

The salinity tolerance of 16 selected isolates was investigated in order to better understand the autecology of different phylotypes using the same media (WC and ASW medium) as used for the long‐term storage of the cultures. Treatments were implemented using triplicates with one control culture that was kept at constant original salinity. Salinity was stepwise increased and decreased, starting from **0**, **23**, or **35** PSU (indicated by *) by removing 10 mL of the 20 mL in each culture flask, followed by resupplying 10 mL of culture media required to reach the desired next concentration (0*—0.5—1—2—4—8—15—25—35—45—60—80—100 [5 isolates]; 0—0.3—0.6—1.3—2.5—5—10—15—23*—35—45—60—80—100—125—150 [2 isolates]; 0–0.3—0.6—1.3—2.5—5—10—15—25—35*—45—60—80—100—125—150 [9 isolates]). Since rhynchomonad cells attach to the bottom of the culture flask with their long posterior flagellum, the final dilution step from 0.3 PSU to freshwater conditions was performed by completely emptying the culture flask, followed by resupplying 20 mL of WC medium. Cultures were kept in climate chambers at 10°C or 20°C, depending on each isolate's preference, based on previous long‐term storage observations. Heat‐killed bacteria (100 μL, *Escherichia coli* ) was supplied during each dilution or concentration step (as long as salinity > 0 PSU) to each culture, including the controls, to exclude the possibility of food scarcity. Culture conditions were evaluated weekly based on cell abundances, motility, and vitality of the observed cells, five points representing very good culture quality and one point meaning death of the culture. If brackish or marine isolates survived the final dilution step, it was performed again to test for prolonged survival potential. Salinity concentrations were measured for one of the three replicates in each dilution or concentration step using a refractometer.

### 2.7 ZooBank Registration

Zoobank registration of present work: urn:lsid:zoobank.org:pub:B68AE649‐58B7‐4D29‐8F4F‐060612F95E35.

## 3 Results

Within this study, we isolated and sequenced 42 new isolates, added four (partial) 28S sequences to previously published isolates and investigated 17 isolates morphologically using high‐resolution video‐enhanced differential interference contrast microscopy and scanning electron microscopy. As a result, nine new species were described. Descriptions were based on the obtained phylogenetic and morphological studies and the results of salinity tolerance experiments.

### 3.1 Phylogenetic Analysis

The 89 available HFCC and GenBank isolate sequences were analyzed using a concatenated (untrimmed) alignment of the SSU and LSU rDNA genes and mapped with a phylogenetic tree based on 4573 nucleotides. The resulting tree is robust with strong support values, especially at the nodes which characterize the different clades. The resulting phylogeny split up into six main clades labeled with Roman numbers. Clade VI branches off from the other five clades as a basal group with the highest p‐distances (SSU rDNA) to all other clades (Figure 3, Table 2). Despite being closely related to each other, the other five clades still exhibit large genetic distances. Pairwise genetic distances (p‐distances), based on 18S rDNA sequences, were used to determine species separations. For this purpose, the long intron in the 18S rDNA sequences was removed for p‐distance calculations. Clade I is mostly composed of deep‐sea isolates from the Atlantic Ocean. However, also isolates from surface waters (HFCC18, HFCC1300), the Pacific Ocean (HFCC830, AF174378), the Mediterranean (HFCC111), the brackish waters of the Baltic Sea (HFCC1420, HFCC1421, HFCC1639, HFCC1641, HFCC1642), and a coastal glacial freshwater lake in Greenland (HFCC826) form part of this clade. All isolates clustering on this branch are genetically identical or show relatively low p‐distances (SSU rDNA) to each other (< 1%; except HFCC148–AF174378: 1.09%; Table S2) and were assigned to *Rhynchomonas metabolita*. Clade II consists of three distinct branches. The first includes six Rhynchomonadidae isolates from marine and brackish water origin and a sequence originally identified as *Cryptaulax* sp. (AY425022). The second branch is composed of five marine deep‐sea isolates (*Rhynchomonas minimamirounga* n. sp.) and the third includes two freshwater isolates (HFCC2052, HFCC2070; *R. reesi* n. sp.) from the Rhine area near Cologne, Germany, which are genetically identical along the 18S rDNA sequence. Isolate HFCC2070 shows p‐distances (SSU rDNA) of 0.86% (HFCC1323), 2.08% (AY425024), and 1.49% (HFCC316) to the other branches (Table S3). Clade III (*Rhynchomonas*) contains nine isolates and consists of two freshwater subclades as well as one marine isolate (AY425023) and one soil isolate (AY998642). The latter two branch basally within this clade and have large p‐distances (SSU rDNA) to the freshwater branch (1.76%–3.15%, Table S4). Clade IV comprises only four marine and brackish water isolates, with HFCC156, HFCC1630, and HFCC1570 clustering together on one branch. Isolate HFCC156 branches basally and shows p‐distances (SSU rDNA) > 3% to the other isolates in this clade (Table S5). Clade V includes a total of 14 isolate sequences. HFCC2100 (*R. mylnikovi* n. sp.) and HFCC17, both originating from freshwater habitats but isolated in different years and from stations more than 2000 km apart from each other, are genetically (18S rDNA sequence) very similar (only one nucleotide difference). HFCC231, isolated from a salt pan, clusters closely to the two freshwater isolates (p‐distance [SSU rDNA] to HFCC2100: 0.49%). The neighboring branch includes three brackish isolates (HFCC1660, HFCC1640, HFCC1657) from the Baltic Sea that are genetically (18S rDNA sequence) almost identical (99.95%–100%) to each other. The remaining eight isolates originate either from the Atlantic Ocean, Caribbean Sea or the North Sea, show p‐distances (SSU rDNA) of 1.07% (Table S6) to each other and all cluster separately, except for isolates HFCC1139 and HFCC1504 that cluster together and show a very small p‐distance (SSU rDNA) (0.2%). With the exception of the three branches containing HFCC2100, HFCC1660, and HFCC1139 that are supported by high bootstrap values, the topology within this clade is not well‐resolved and shows weak bootstrap support on several nodes. Clade VI only contains three isolates from marine and brackish water origin and diverges at the basis of the other clades. It shows large p‐distances (SSU rDNA) (6.33%–8.11%, see Table 2) to Clades I–V. The three isolates of this clade belong at least to separate species highlighted by the large p‐distances (SSU rDNA) to each other (2.76%–4.53%, see Table S7). Several sequences included in the phylogenetic tree have a long intron between nucleotide positions of about 435 and 989 (see Figure S1). The introns have a length of approximately 500 nucleotides, but differ slightly in length. Among the 46 sequences of Clade I, 33 sequences show such an intron. The 33 introns share most nucleotide positions with each other, however not all. In Clade II, 4 out of 14 sequences have a similar intron. With the exception of four positions, the introns for HFCC173, HFCC196, and HFCC171 are identical. The intron of HFCC147 deviates strongly from the other three. In Clade III and IV, no such introns were found. In Clade V, only HFCC231 has a long intron. In Clade VI, only HFCC99 has a long intron. For both clades, introns strongly deviate from one another and from all other introns in the other clades. The existence of this long intron has been reported before for isolate HFCC99 (Scheckenbach et al. 2005). Representatives of Clade VI lack short introns of about 10 nucleotides length starting at position 1348 of the alignment (Data S1). The topology in Clades I and V differs slightly for the ML and BI trees. This does not concern decisive nodes that determine the clustering of the six main clades, but indicates that the topologies of deeper branches are not well‐resolved in several cases. In four instances, in Clades I and V, isolates from freshwater environments clustered together with isolates originating from brackish or marine habitats. In Clade I, isolate HFCC826, isolated from a freshwater lake near Ilulissat, Greenland, represents an exception from the other freshwater isolates in the tree. It clusters together with many highly similar isolates from marine and brackish habitats. However, the isolate has been sampled within 20 km distance to marine waters and several marine species have been found in this relatively recently formed glacial lake (unpublished data). The p‐distances calculated for the 28S rDNA confirm the 18S results (see Table S8). Apart from comparisons among *Clade I Rhynchomonas* spp., the pairwise genetic distances between the individual isolates even exceed the 18S p‐distances. The untrimmed stand‐alone 18S rDNA phylogeny confirms the results obtained from the concatenated phylogeny (see Figure S2). The clades and subclades (species) cluster in the exact same way, with almost identical bootstrap support. Additionally, the stand‐alone 18S rDNA phylogeny from the trimmed alignment shows highly similar clustering, however often with slightly lower bootstrap support (see Figure S3). The individual isolates remain all in the previously described six clades and all isolates that are characterized as the individual species still cluster together with high bootstrap support.

**FIGURE 3:**
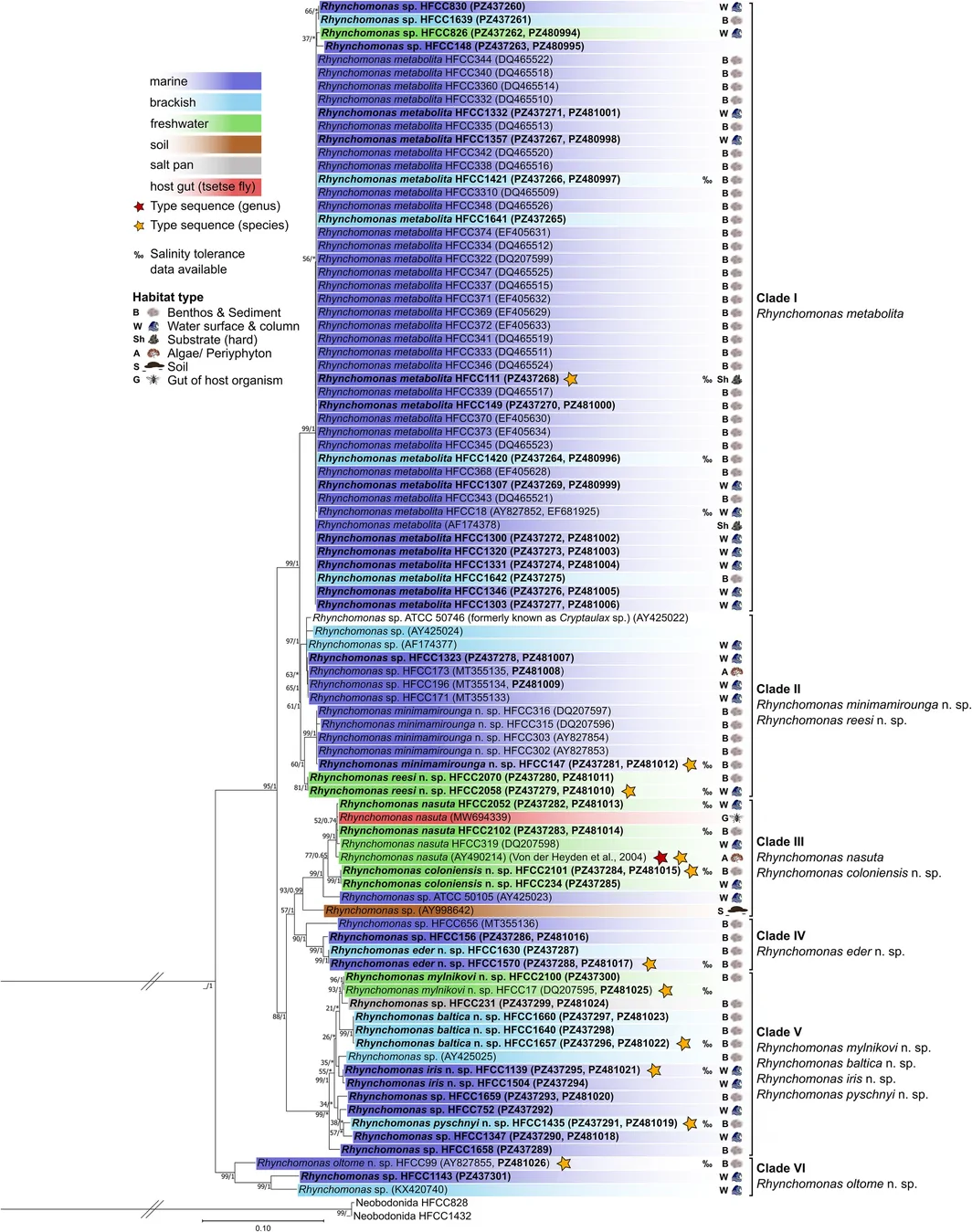
Phylogenetic tree of rhynchomonads based on SSU rDNA and partial LSU rDNA sequences. Bootstrap values from maximum likelihood analysis (1000 replicates) and Bayesian inference analysis (2,710,000 generations) are depicted at the nodes. Differences in the topology between the two methods are indicated by “*.” Isolates from this study are marked in bold. Red stars indicate the type isolate for the genus.

**TABLE 2:** P‐distances (SSU rDNA) of selected Rhynchomonadidae isolates from all six clades calculated with the pairwise alignment function from BioEdit. Identifiers refer to internal HFCC database or GenBank accession numbers. The respective alignment on which the p‐distance calculation is based is given in the Supporting Information.

Isolate		HFCC 111	HFCC 147	HFCC 2070	AY490 214	HFCC 2101	AY998 642	HFCC 656	HFCC 1570	HFCC 1657	HFCC 1139	HFCC 99
	Clade	1	2	2	3	3	3	4	4	5	5	6
HFCC147	2	1.98%										
HFCC2070	2	1.89%	1.54%									
AY490214	3	5.17%	4.54%	5.05%								
HFCC2101	3	5.01%	4.94%	4.69%	1.90%							
AY998642	3	4.42%	3.75%	3.71%	3.05%	3.12%						
HFCC656	4	4.91%	4.67%	5.07%	4.35%	5.21%	4.27%					
HFCC1570	4	4.98%	3.91%	3.93%	3.96%	4.51%	3.15%	3.45%				
HFCC1657	5	5.51%	5.31%	4.85%	4.85%	5.20%	4.71%	5.06%	3.97%			
HFCC1139	5	5.20%	5.05%	5.16%	4.55%	4.77%	4.24%	5.24%	4.29%	1.48%		
HFCC99	6	6.78%	6.99%	6.95%	6.94%	6.85%	6.61%	7.82%	7.12%	6.93%	6.33%	
HFCC1143	6	7.06%	7.26%	7.65%	7.41%	7.63%	7.44%	8.11%	7.80%	7.92%	7.46%	4.53%

### 3.2 Morphology

All morphologically investigated isolates share very similar characteristics: a colorless flattened oval shaped cell body, a very ductile proboscis at the anterior end of the cell that it uses for searching food particles in an up and down sweeping motion, a long posterior flagellum (with an acroneme at the end) arising in the flagellar pocket (located at the anterior end of the cell) and attached to the surface that is used for gliding on the substrate, and a short anterior flagellum arising in the flagellar pocket, which is held close to the proboscis and only visible in few instances. The morphological measurements of each isolate are given in Figure 4 and Table S1. The ratio of the cell size to the length of the posterior flagellum is very stable within each isolate but differs greatly between the different isolates, including between isolates of the same species level. Some isolates (e.g., HFCC99, HFCC1139, HFCC1657, HFCC2058, HFCC2102) exhibit a rounder shape (lower length:width ratio) while others (e.g., HFCC1420, HFCC1570) are more elongated. The acroneme of the posterior flagellum occasionally changes in length for individual cells as it is, for instance, the case for isolate HFCC148 (*Rhynchomonas metabolita*). The length of the flagellum without the acroneme was similar in both analyzed frames of the same cell (Figure 5D: 11.3 μm, E: 11.2 μm), but the acroneme length differed a lot (Figure 5D,E). The electron micrographs of the analyzed 16 isolates show the basic cell characteristics (anterior flagellum, long posterior flagellum, paraxial rod, acroneme, and proboscis) of rhynchomonads that have been described by previous authors. Suspected cyst formation could be observed for isolates HFCC18, HFCC2101, and HFCC2102 (Figure 5A–C). Mastigonemes on the flagella were not visible in our SEM preparations. The uptake of both bacilli and cocci bacterial cells was observed (Figure 5F,G). Available DIC and SEM micrographs are summarized for the different isolates in Figures 5, 6, 7, 8, 9.

**FIGURE 4:**
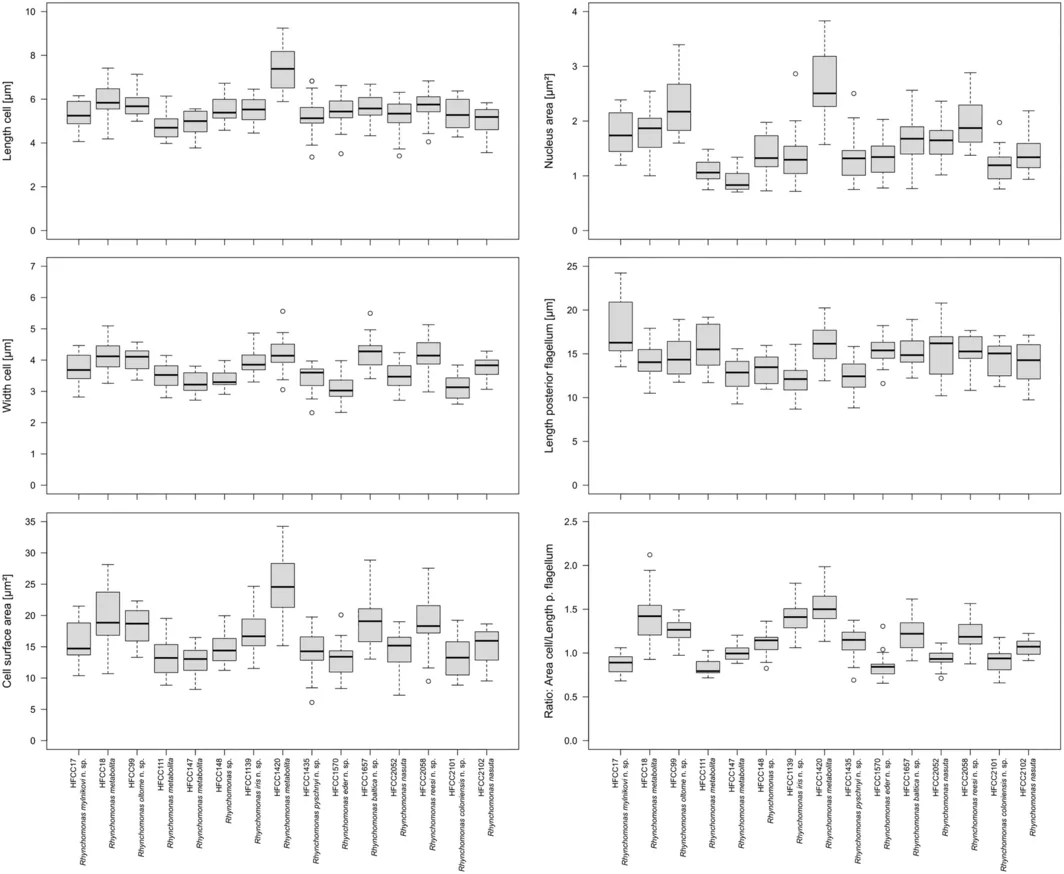
Characteristic dimensions of analyzed rhynchomonad isolates derived from live observations (DIC micrographs).

**FIGURE 5:**
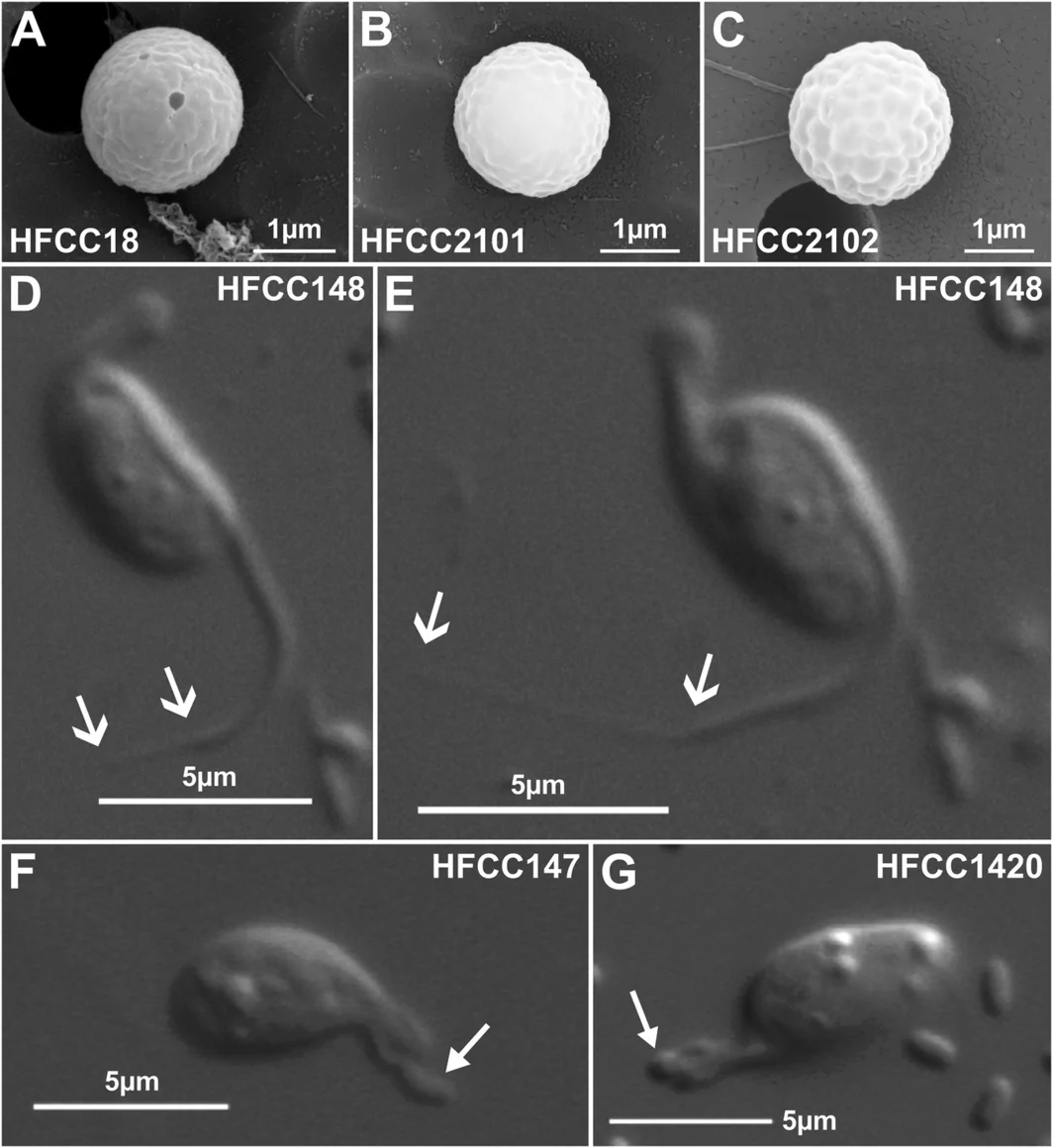
SEM micrographs of (potential) cysts (A–C) and DIC micrographs of retracted (D) and extended (E) acroneme (same cell) and uptake of bacilli (F) and cocci (G) bacteria of different rhynchomonad isolates.

**FIGURE 6:**
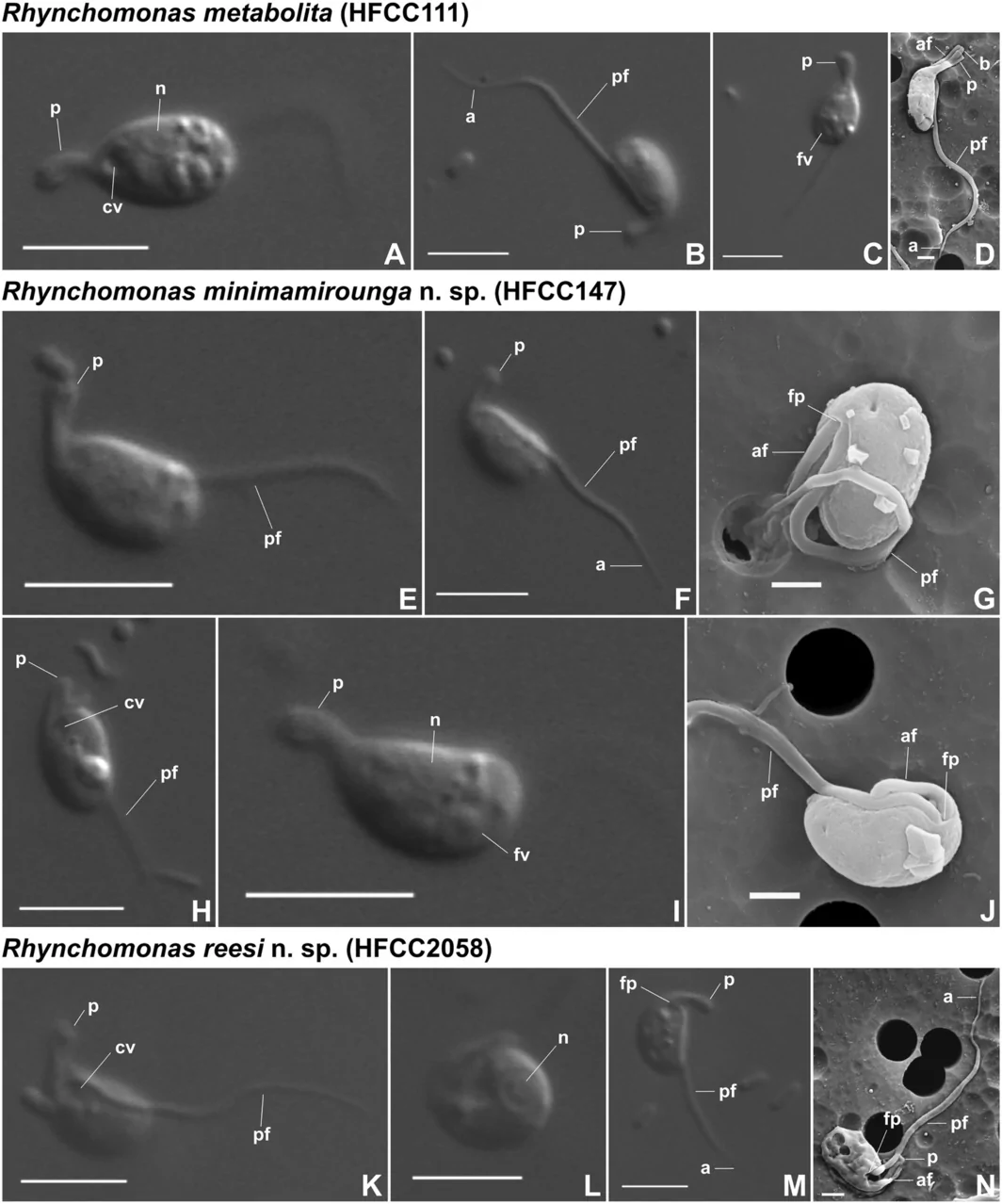
DIC and SEM micrographs for Clades I and II (A–D: *R. metabolita*, E–J: *R. minimamirounga* n. sp.,K–N: *R. reesi* n. sp.). Scale bars for micrographs equal 5 μm (DIC) and 2 μm (SEM). The proboscis (p), posterior flagellum (pf), acroneme (a), anterior flagellum (af), nucleus (n), contractile vacuole (cv), food vacuoles (fv), and flagellar pocket (fp) are indicated. The uptake of a bacterial cell (b) can be seen in (D).

**FIGURE 7:**
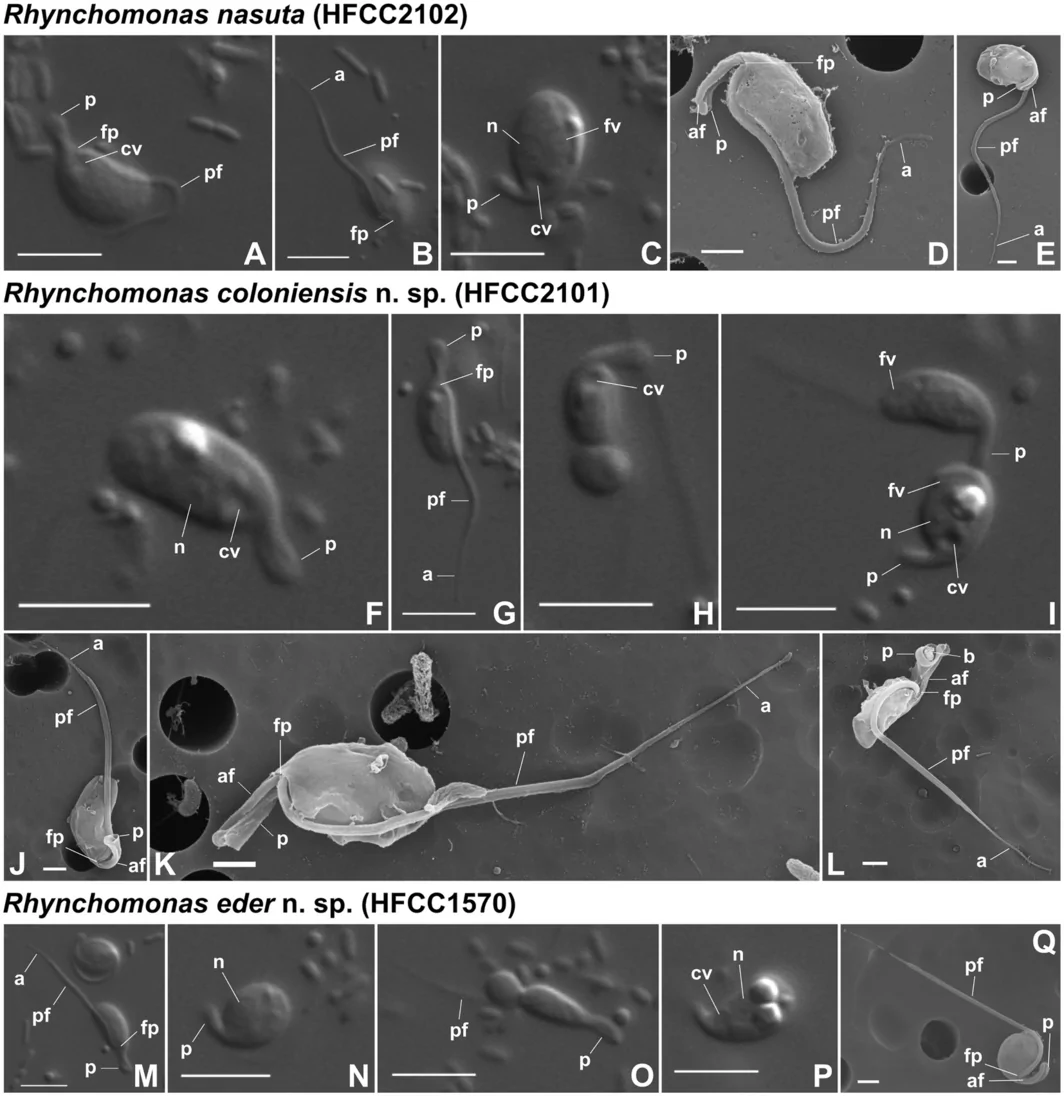
DIC and SEM micrographs for Clades III and IV (A–E: *Rhynchomonas nasuta*, F–L: *R. coloniensis* n. sp., M–Q: *R. eder* n. sp.). Scale bars for micrographs equal 5 μm (DIC) and 2 μm (SEM). The proboscis (p), posterior flagellum (pf), acroneme (a), anterior flagellum (af), nucleus (n), contractile vacuole (cv), food vacuoles (fv), and flagellar pocket (fp) are shown. The uptake of a bacterial cell (b) can be seen in (L). The metabolic character of the cells can be seen in (H, O). The cell thickness can be seen in (L).

**FIGURE 8:**
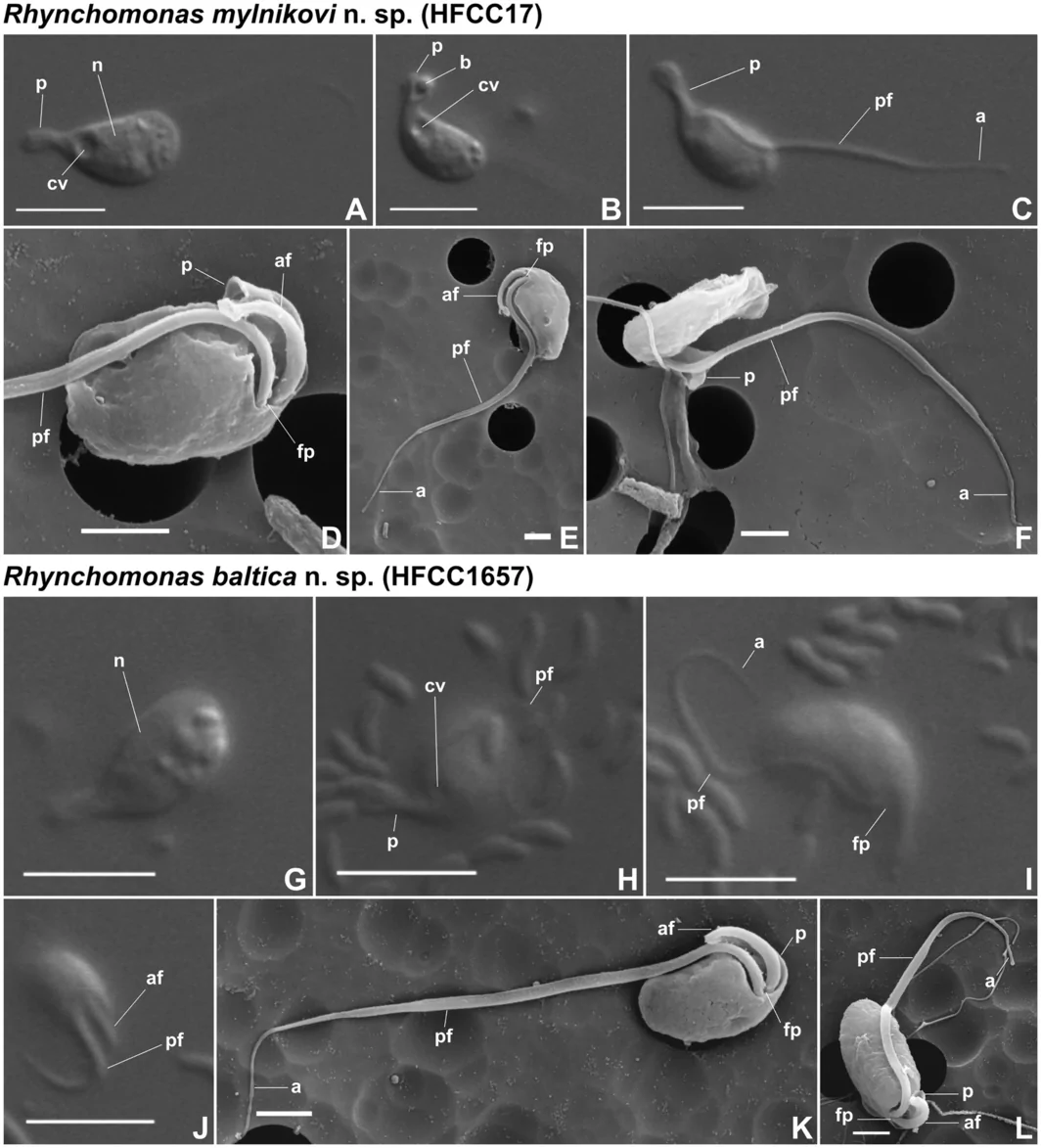
DIC and SEM micrographs for Clade V (A–F: *Rhynchomonas mylnikovi* n. sp., G–L: *Rhynchomonas baltica* n. sp.). Scale bars for micrographs equal 5 μm (DIC) and 2 μm (SEM). The proboscis (p), posterior flagellum (pf), acroneme (a), anterior flagellum (af), nucleus (n), contractile vacuole (cv), and flagellar pocket (fp) are shown. The uptake of a bacterial cell (b) can be seen in (B). The cell thickness can be seen in (F).

**FIGURE 9:**
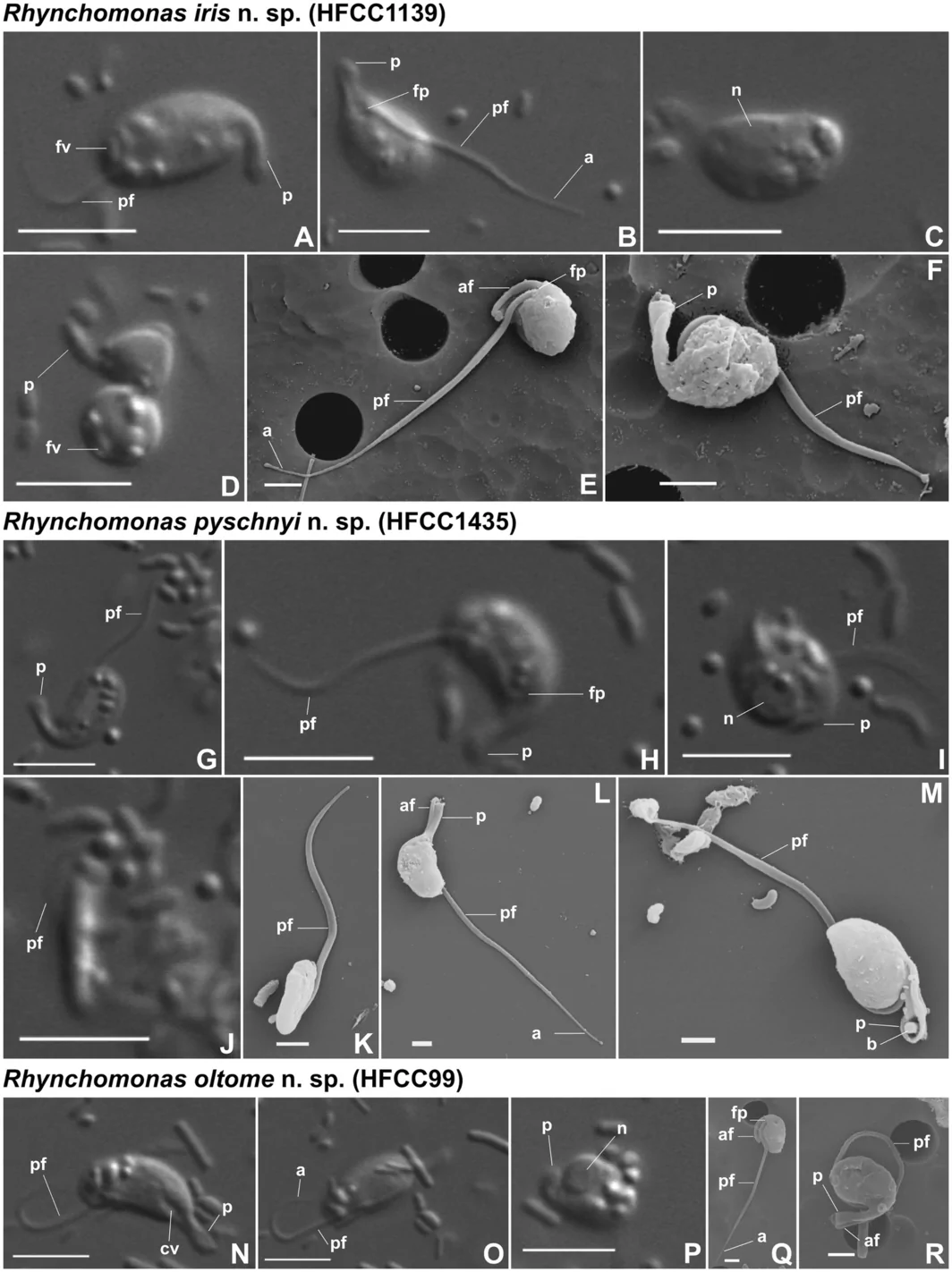
DIC and SEM micrographs for Clade V (A–F: *Rhynchomonas iris* n. sp., G–M: *R. pyschnyi* n. sp.) and Clade VI (N–R: *R. oltome* n. sp.). Scale bars for micrographs equal 5 μm (DIC) and 2 μm (SEM). The proboscis (p), posterior flagellum (pf), acroneme (a), anterior flagellum (af), nucleus (n), contractile vacuole (cv), food vacuoles (fv), and flagellar pocket (fp) are shown. The uptake of a bacterial cell (b) can be seen in (M). The metabolic character of the cells can be seen in (D). The cell thickness can be seen in (J, K).

### 3.3 Salinity Tolerance Experiment

Results obtained from studies on salinity tolerance showed large differences in the tolerance ranges of the different isolates, especially when comparing isolates from marine/brackish habitats with those from freshwater habitats (Figure 10, Figure S4). Isolates originating from freshwater environments seem to be stenohaline (lowest tolerance range); however, all are able to tolerate certain levels of brackish conditions. Isolates HFCC2052 and HFCC2102, both molecularly identified as *Rhynchomonas nasuta*, show the same tolerance levels. Isolate HFCC2101 (*Rhynchomonas coloniensis* n. sp.) shows the lowest salinity tolerance (max. 5 PSU) of any tested freshwater isolate and isolate HFCC2058 (*Rhynchomonas reesi* n. sp.) exhibited the highest tolerance with all replicates surviving a salinity of 27 PSU. Most brackish isolates, as expected, tend to have a higher range of tolerance. For instance, HFCC1657 (*Rhynchomonas baltica* n. sp.), isolated from a salinity of 12 PSU, tolerated up to 86 PSU (range of reproduction: 0–50 PSU; dense and active culture at 0 PSU), while HFCC1420 and HFCC1421 (*Rhynchomonas metabolita*), isolated from a salinity of 19 PSU, tolerated more than 100 PSU (range of reproduction: 1.3–87 PSU; almost no living cells at freshwater conditions). Within the marine isolates of *Rhynchomonas* spp., isolates HFCC99 and HFCC1570 show much lower tolerance ranges than isolates HFCC18, HFCC111, and HFCC147. Notably, all isolates belonging to the Clades I and II, namely HFCC2058, HFCC1420, HFCC1421, HFCC18, HFCC111, and HFCC147, exhibit higher tolerance ranges than other rhynchomonad isolates from environments with the same or similar salinity concentrations. All replicates from the marine (HFCC18, HFCC111, HFCC147) and brackish (HFCC1420 and HFCC1421) isolates belonging to the two *Rhynchomonas* Clades I and II were able to survive salinities well over 90 PSU, exhibiting the highest salinity tolerances among all tested isolates. The four isolates of *Rhynchomonas metabolita* (HFCC18, HFCC111, HFCC1420, and HFCC1421) show relatively similar tolerance ranges, despite originating from either brackish or marine waters; this species might be characterized as a euryhaline species within the *Rhynchomonas* species complex. The largest range of tolerance was found for *Rhynchomonas minimamirounga* n. sp. isolated from the deep sea of the Eastern South Atlantic tolerating salinities from 2 to 135 PSU (Figure 10).

**FIGURE 10:**
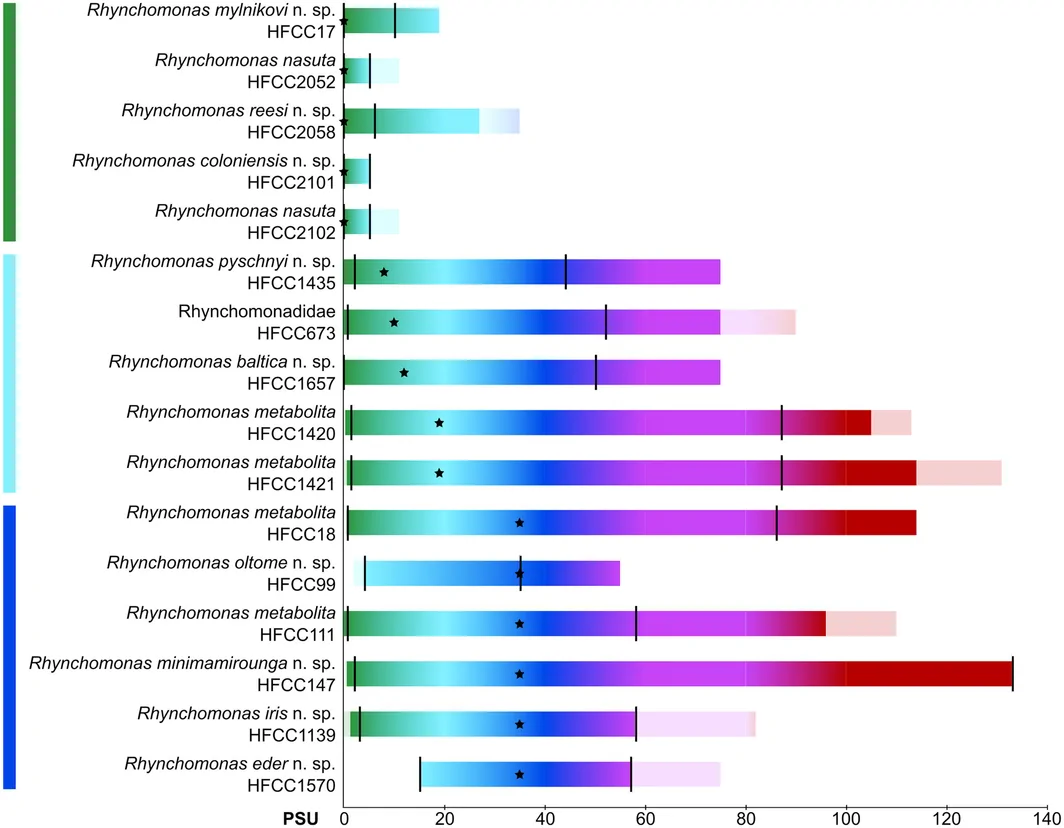
Salinity tolerance of 16 isolates of Rhynchomonadida. Stars indicate the salinity of the sampling location (5 freshwater, 5 brackish, 6 marine isolates). The colored sidebars on the left indicate freshwater (green), brackish (light blue), and marine (dark blue) origin. The range between black dashes indicates the comfort zone of each isolate at which reproduction was observed (at least 3.5 points average and no quality score lower than three points among the three replicates). Bright colors indicate the survival range of all replicates. Transparent range indicates at least one surviving replicate.

## 4 Discussion

### 4.1 Neotype Designation for *Rhynchomonas nasuta*

Stokes (1888) described the first specimen from a freshwater pond with standing water that contained decaying *Sphagnum* moss. Unfortunately, the original isolate is not available anymore and, to our knowledge, no renewed isolation from the original habitat has been conducted. Despite its cosmopolitan distribution and frequent mentioning in studies, *Rhynchomonas* isolates have scarcely been isolated and sequenced along their full‐length SSU rDNA gene, especially from continental aquatic habitats. For the United States of America, from where it was originally described by Stokes, only two brackish isolates from Chesapeake Bay were publicly available (AY425024, AF174377). Therefore, isolate AY490214 (Von der Heyden et al. 2004) isolated from Priest Pot, Cumbria, England is designated as the neotype for *Rhynchomonas nasuta*. Priest Pot (Esteban et al. 2012; Folkard et al. 2007) is a very well‐investigated eutrophic pond which served as a model for protistological studies (Finlay 1998), resembles probably best the habitat conditions of the originally discovered isolate. Additionally, AY490214 isolated by Von der Heyden et al. (2004) clusters together with other isolates from freshwater habitats from Germany and the Central African Republic, showcasing the wide geographic distribution of the neotype. The designation is furthermore a tribute to the authors and their extensive contributions to protistology.

### 4.2 Morphological Data

Based on the videos and images obtained by light microscopy and electron microscopy, no clear distinctions of morphological characteristics could be made for the investigated isolates. All isolates share the same basic characteristics with an ovoid flattened cell body, an anterior proboscis and short flagellum attached to the proboscis, a long posterior flagellum attached to the surface and a nucleus located in the anterior part of the cell body. The ratio of the cell area to the length of the posterior long flagellum was very stable within the isolates. However, no conclusive pattern could be obtained that would enable a clear and significant distinction between the identified clades or species based on the determined parameters. Furthermore, it has been observed that morphological characteristics of protists such as the cell size and volume vary with dietary conditions (Christaki et al. 2005) and environmental conditions (Atkinson et al. 2003; Mulot et al. 2017) or even without any changing parameters (Medioli et al. 1987), showcasing the potential phenotypic plasticity of these organisms. This plasticity makes it difficult to distinguish cryptic heterotrophic nanoflagellates solely based on morphological measurements and requires taking autoecological and molecular data into account. Locomotion patterns are influenced by external abiotic parameters (e.g., temperature, Chahal et al. 2018). The metabolic rhynchomonads are forced to change direction or shape frequently by adjusting the cell length:width ratio, which makes it difficult to base descriptions of rhynchomonads on morphological characters. Future detailed ultrastructural studies may show that some of the genotypes described here can be separated using detailed morphological characters as it was possible for closely related species of the bicosoecid *Caecitellus* (Hausmann et al. 2006), however, for routine differentiation of rhynchomonads, genotypic characterizations might be much easier and more practical.

### 4.3 Ecology

Representatives of the *Rhynchomonas* morphotype have been identified from all types of ecosystems distributed worldwide. This is also exemplified by the 216 georeferenced records of this morphotype (Figure S5). Isolates belonging to the same species only occasionally cluster together based on their geographical origin. Examples include *Rhynchomonas reesi* n. sp. (HFCC2058, HFCC2070) from the Rhine floodplains close to the Dutch border, *Rhynchomonas coloniensis* n. sp. (HFCC234, HFCC2101) from the River Rhine in Cologne and *Rhynchomonas baltica* n. sp. (HFCC1640, HFCC1657, HFCC1660) from the western Baltic Sea. Contrary, isolates belonging to the species *Rhynchomonas pyschnyi* n. sp., *Rhynchomonas eder* n. sp., *Rhynchomonas nasuta*, or *Rhynchomonas metabolita*, latter includes 46 isolates from very different world regions, are not sharing similar geographical origins. Undersampling may be an explanation for the lack of isolates within seemingly geographic restricted species and could disprove a high degree of endemism within the rhynchomonads, which might be excluded by including metabarcoding data. Alternatively, the existence of locally adapted as well as cosmopolitan species could serve as an explanation as it was found for other groups of nanoflagellates (e.g., Hohlfeld et al. 2023; Rybarski et al. 2021). In spite of obvious limitations such as undersampling of various world regions and habitats, certain genotypes have been isolated frequently and seem to be distributed much more widespread than others. Despite frequent detections in soil ecosystems (Domonell et al. 2013; Ekelund and Patterson 1997; Frederiksen et al. 2001), only one soil isolate (AY998642) has been isolated and sequenced (Koch and Ekelund 2005), showcasing the requirement for further studies on rhynchomonads in other environments. Overall, rhynchomonads examined in this study exhibited a broad salinity tolerance from 0 to 135 PSU (Figure 10) confirming results from previous studies (e.g., Mylnikov 1983; Patterson and Simpson 1996). Similarly, it has been shown experimentally (Priya et al. 2008, 2019) and regarding their distribution in the environment (Bernard et al. 2000; Edgcomb et al. 2011) that certain rhynchomonad isolates are able to withstand or grow under anoxic conditions. Others seem to survive only temporary anoxia through passive stages or do not survive at all (Table 3). Furthermore, marine isolates of different origin show large differences in pressure tolerance levels (Živaljić et al. 2018). The strongly deviating physiological barriers of the different isolates regarding a variety of abiotic factors make it clear that the isolates might not belong to the same species. Rather, the *Rhynchomonas* morphospecies complex has to be understood as a highly successful morphotype that is genetically and physiologically adapted to varying ecological niches, carrying out similar ecological functions. This argument has also been brought forward for *Spumella*‐like phyloclades and their ecophysiological adaptations (Stoeck et al. 2008) or the cryptic *Neobodo designis* complex (Koch and Ekelund 2005). While a distinction by ecological traits can be derived from the abiotic factors mentioned above, it is most robustly backed by the salinity tolerances of the individual subclades. The salinity tolerance experiments carried out within this study as well as previous studies (Table 3) show clearly that freshwater isolates are consistently stenohaline and only able to tolerate a narrow range of brackish conditions, with ideal growth conditions at low salinities. This stands in stark contrast to other species (e.g., *Rhynchomonas metabolita*) that are capable to thrive under high salinities, but do not tolerate freshwater conditions. Certain marine isolates, like *Rhynchomonas eder* n. sp. (HFCC1570), represent stenohaline marine species and do not tolerate freshwater or slightly brackish conditions and have no overlapping tolerance range with certain species from freshwater origin such as *Rhynchomonas nasuta* (HFCC2052, HFCC2102) or *Rhynchomonas coloniensis* n. sp. (HFCC2101). The clear phylogenetic clustering of isolates from freshwater origin and the absence of marine isolates from these monophyletic subclades for the species *Rhynchomonas reesi* n. sp., *Rhynchomonas nasuta*, *Rhynchomonas coloniensis* n. sp., and *Rhynchomonas mylnikovi* n. sp. supports the taxonomic separation on the basis of their different physiology. The evolutionary adaptation to freshwater habitats of the individual taxa could have slowly led to the diminishing capacity to tolerate higher salinities, and vice versa for marine representatives within the Rhynchomonadidae. Despite the complexity of evolutionary changes that are required for physiological adaptations to overcome the salt barrier, transitions have occurred several times in specific clades, for instance in dinoflagellates, indicating that the salt barrier might be less strong than so far suspected, as noted by Jamy et al. (2022). The phylogenetic and salinity tolerance results from this study affirm these notions. The adaptation to freshwater or marine conditions seems to have occurred several times and can be seen in the Clades I, II, III, and V.

**TABLE 3:** Tolerances of rhynchomonads with regard to hydrostatic pressure, high salinities, and anoxia reported from literature and own observations. The results from the salinity tolerance experiment of this study are not included in this table.

Isolate	Parameter	Result	Source
BSZ1 (AF174378*) R. metabolita*	Pressure	Tolerance up to 300 bar	Atkins et al. (1998)
CBR1 (AF174377) *R*. sp.	Pressure	Tolerance up to 300 bar	Atkins et al. (1998)
HFCC18 *R. metabolita*	Pressure	Tolerance up to 550 bar	Živaljić et al. (2018)
HFCC99 *R. oltome* n. sp.	Pressure	Tolerance up to 300 bar	Živaljić et al. (2018)
HFCC147 *R. minimamirounga* n. sp.	Pressure	Tolerance up to 550 bar	Živaljić et al. (2018)
HFCC149 *R. metabolita*	Pressure	Tolerance up to 300 bar	Živaljić et al. (2018)
HFCC171 *R*. sp.	Pressure	Tolerance up to 660 bar	Živaljić et al. (2018)
HFCC173 *R*. sp.	Pressure	Tolerance up to 670 bar	Živaljić et al. (2018)
Not further specified	Salinity	Tolerance up to 10 PSU (fw isolate)	Ekelund (2002)
Not further specified	Salinity	Tolerance up to 18 PSU (fw isolate)	Arndt et al. (2000)
Not further specified	Salinity	Growth up to 15 PSU (fw isolate)	Mylnikov (1983)
Free‐living cells	Salinity	Observed in environment at 150 PSU	Patterson and Simpson (1996)
HFCC1660 *R. baltica* n. sp.	Anoxia	Active cells after 72 h anoxia	Unpublished data
HFCC1657 *R. baltica* n. sp.	Anoxia	Survival as resting stage	Unpublished data
HFCC1420 *R. metabolita*	Anoxia	Survival as resting stage	Unpublished data
HFCC1435 *R. pyschnyi* n. sp.	Anoxia	Survival as resting stage	Unpublished data
HFCC1421 *R. metabolita*	Anoxia	No survival	Unpublished data
HFCC1659 *R*. sp.	Anoxia	No survival	Unpublished data
Not further specified	Anoxia	Survival & growth	Priya et al. (2008, 2019)

### 4.4 Molecular Data and Phylogeny

The isolates characterized in this study almost double the number of sequences available for the highly successful and widespread *Rhynchomonas* morphotype. Isolates from freshwater, brackish, and marine waters have been included and revealed new phylogenetic lineages. The cryptic character of the *Rhynchomonas* morphotype complex has already been recognized before (Birky et al. 2010; Callahan et al. 2002; Domonell et al. 2013; Scheckenbach et al. 2005; Von der Heyden et al. 2004), highlighting the urgently needed phylogenetic and taxonomic revision of this cryptic complex of species. As pointed out by Boenigk et al. (2012) and others (Azovsky et al. 2016; Caron 2013), taxonomic revisions should be based on different methods, namely by combining morphological, ecophysiological and molecular techniques. This study tried to follow this line and analyzed the abovementioned characteristics for all isolates analyzed in this study. In the absence of clear physiological and morphological clustering of the rhynchomonads in our study, we followed Boenigk et al. (2012), differentiating species based on genotypical properties, in this case with the help of autoecological data. This is also in line with the notions by Leander (2023) on the morphological stability of phagotrophic flagellates over enormous phylogenetic distances (morphostasis) and the resulting importance of genomic information for taxa differentiation. Taxonomic reclassifications of cryptic or hardly‐distinguishable morphospecies complexes are always complicated. New descriptions of species and genera within such complexes have repeatedly and increasingly become necessary for heterotrophic protists of different phylogenetic origin since the onset of phylogenetics and phylogenomics, for instance for the genera *Cunea* (Kudryavtsev and Pawlowski 2015; Kudryavtsev and Volkova 2020), *Euglenaformis* (Bennett et al. 2014), *Discoplastis* (Łukomska‐Kowalczyk et al. 2021), and *Caecitellus* (Hausmann et al. 2006). The principle has also been applied for the species delineation of the morphologically highly similar species of placidids and colorless chrysomonads (Pfandl et al. 2009; Pietsch et al. 2022; Rybarski et al. 2021). Many more taxa await revision to better understand the true diversity of tiny morphostatic protists (Leander 2023). It should be noted that molecular, ecological and behavioral information and differences between isolates are of even greater value for truly morphologically morphostatic groups, likely the rhynchomonads, compared to other groups of heterotrophic nanoflagellates, where distinguishable morphological characteristics might be very small but present. Considering the molecular and phylogenetic results obtained throughout this study as well as arguments on large genetic variations and physiological barriers brought forward in previous phylogenetic and autoecological studies on the Rhynchomonadidae and other groups within the Metakinetoplastina (Azovsky et al. 2016; Callahan et al. 2002; Koch and Ekelund 2005; Scheckenbach et al. 2006), the current morphospecies *Rhynchomonas nasuta* can no longer be maintained as one species. Therefore, the *Rhynchomonas* morphotype is split into 11 distinct species, which are supported through high bootstrap support for the six clades and the subclades (species level). A neotype (AY490214) is designated for the existing *Rhynchomonas nasuta*. As there is no standard regarding *p* values and its application for species differentiation among protists, we suggest the combination of clear phylogenetic clustering and the use of threshold values as described below, excluding long introns. All following values are based on comparisons of the same available marker region and calculations were carried out in the same manner. P‐distances (SSU rDNA) between the clades range between 3.15% and 8.11% (Table 2). The differentiation of isolates on species level was based on the clustering in the phylogenetic analysis and calculated p‐distances (SSU rDNA) within the clades, which always fall short of 1%. We abstained from assigning isolates HFCC830, HFCC1639, HFCC826, HFCC148 (all Clade I), and HFCC231 (Clade V) to concrete species due to their slight deviations (clustering and genetic distances) to the other closely related described species. This also seems appropriate when considering prior claims that two *Rhynchomonas* isolates with a p‐distance of 1.9% probably represent distinct species (Callahan et al. 2002) or comparing the p‐distances in better described species complexes within the Metakinetoplastina. For instance, the genetic distance between the three species *Cryptobia catastomi*, *Cryptobia bullocki*, and *Cryptobia salmositica* was calculated (BioEdit pairwise alignment function) to be less than 1.6% (isolates AF080226, AF080225, AF080224 from Wright et al. 1999). The genetic distance between *Dimastigella mimosa* and *Dimastigella trypaniformis* with contrasting lifestyles (parasitism vs. free‐living; Štolba et al. 2001), the currently only other genus within the Rhynchomonadidae, only amounts to 0.1%–0.3% (based on the 18S rDNA of isolates AF208882, DQ207576, AY753598, X76495 from the phylogeny of Belyaev et al. 2022). With regard to the overall genetic diversity in a cryptic morphotype clade, the *Bodo saltans* complex, a mean p‐distance of 3.82% was calculated for this clade, which is very similar to the mean p‐distance calculated for the *Rhynchomonas* complex (3.55%). However, in contrast to the *Rhynchomonas* complex, the *Bodo saltans* complex already includes two other species, *Bodo edax* and *Bodo uncinatus* that cluster closely with other *Bodo saltans* isolates. In order to further support the division into six clades, molecular signature sequences (motifs) in the SSU rDNA were identified (Figure 11). There are relatively few motifs for Clades III and IV due to a high degree of intraclade variability. In Clade V, we describe isolates HFCC2100, HFCC17 (*Rhynchomonas mylnikovi* n. sp.), HFCC1660, HFCC1640, HFCC1657 (*Rhynchomonas baltica* n. sp.), HFCC1139, HFCC1504 (*Rhynchomonas iris* n. sp.), and HFCC1435 (*Rhynchomonas pyschnyi* n. sp.) as new species, but abstain from designating any other isolate to the newly described species due to unresolved branching within the clade and relatively high p‐distances (SSU rDNA) (0.98%–2.1%). We are aware that the classification of all isolates into six clades might not represent the actual final diversity of the highly diverse rhynchomonads, though it might depict best the current available information within the Rhynchomonadidae. In particular, the isolates in clade VI and the soil isolate AY998642 in clade III show great genetic differences to the next closest related isolates, providing room for the discovery of further closely related species. The publicly available data on the molecular diversity of rhynchomonads is still scarce and most often limited to the 18S rDNA. Wherever possible, we sequenced the 28S rDNA to build a more robust phylogeny. In the future, we recommend to consistently sequence the SSU and LSU in order to construct solid concatenated trees, and possibly even include further genes to build a more extensive multigene phylogeny, as it is nowadays often done for other protist taxa.

**FIGURE 11:**
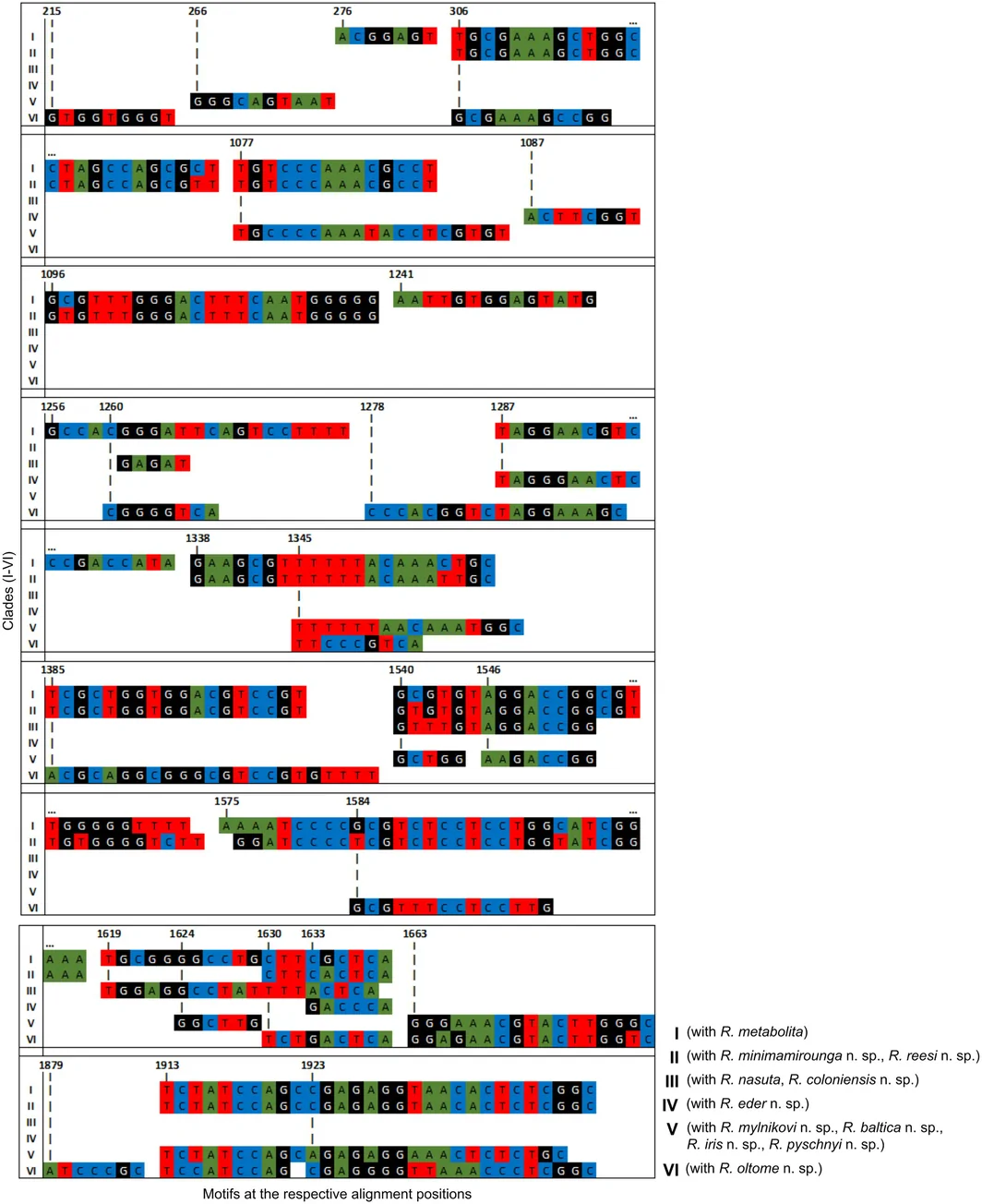
Diagram illustrating synapomorphic motifs within the 18S rDNA across the six clades, highlighting dissimilarities between the sequence clusters. The numbers above each motif represent the start position of the motif within the original alignment of this study (Supporting Information). Variations in the motifs underscore evolutionary relationships and distinctions.

## 5 Taxonomic Summary

*Rhynchomonas nasuta* was originally described as *Heteromita nasuta* by Stokes (1888) and then transferred to the new genus *Rhynchomonas* by Klebs (1892). Both descriptions were short and only described basic characteristics of the species. Lohmann (1912, 1920) described four new species, which have later been reassigned by Schiller (1932) to the taxa *Pronoctiluca pelagica* (before *Rhynchomonas marina*; Fabre‐Domergue 1889; Guiry 2002), *Pronoctiluca spinifera* (before *Rhynchomonas spinifer*; Guiry 2012) and *Pronoctiluca acuta* var. *curvata* (before *Rhynchomonas acuta* and *Rhynchomonas curvata*; Molinari Novoa and Cowan 2023; Molinari Novoa and Murphy 2003). Similarly, *Rhynchomonas mutabilis* described by Griessmann (1913) was reassigned to the Thecamonadidae (*Thecamonas mutabilis*) by Larsen and Patterson (1990). Bělař (1916) amended the genus *Rhynchomonas* and species ( *R. nasuta* ) characterizations with detailed descriptions of the cell structure, the mentioning of cyst formation, movement pattern, cell division, and first phylogenetic relationships. Another proposed species description of *Rhynchomonas offendens* (Skuja, 1956, cited in Larsen and Patterson 1990) has later been rejected due to the absence of the characteristic snout (proboscis) in the described organism (Larsen and Patterson 1990). An important amendment to the *Rhynchomonas nasuta* morphotype has been carried out by Swale (1973) who closely studied morphological characteristics and internal cell structures of the proboscis, flagellar pit, flagella (with mastigonemes) contractile vacuole, food vacuoles, nucleus und nucleolus, kinetoplasts, mitochondrion, Golgi body, cell membrane, microtubular bands, lumen as well as cell movements, flexibility and the feeding process using preparations of fixed and living cells for light and electron microscopy. The latest species description for the genus *Rhynchomonas* was carried out by Pshenin (1964). His *Rhynchomonas metabolita* was isolated from benthic deep‐sea samples from the Mediterranean Sea. The author noted the similarity in size, morphology, and further characteristics to *Rhynchomonas nasuta*, and based the new description on the highly flexible and metabolizing cells of this isolate. The formation of cysts was also observed. However, this metabolic, shape‐changing capacity is not restricted but rather part of the morphological plasticity of all isolates (i.e., compare Figures 5, 6, 7, 8, 9). The distinguishability of the two species, *R. nasuta* and *R. metabolita*, cannot be confirmed solely based on the cell flexibility, as already noted previously (Burzell 1973; Larsen and Patterson 1990). According to the thorough morphological description of the isolate (Pshenin 1964) and the amendment of *Rhynchomonas metabolita* by Burzell (1973) who described the proboscis‐cytopharynx complex based on electron micrographs, *R. metabolita* is considered as a valid species.

Assignment: Eukaryota, Discoba, Euglenozoa, Kinetoplastea, Metakinetoplastina, Neobodonida, Rhynchomonadidae.

## 6 Taxonomy of Novel Species

### 6.1 *Rhynchomonas* Klebs 1892, n. emend. Wagenhofer, Nitsche & Arndt

**Diagnosis**. Free‐living, colorless rhynchomonad flagellate, cells are biflagellated, both flagella arise in the flagellar pocket at the anterior end. The longer posterior flagellum trails behind the cell and is used for attaching and gliding on the substrate. The shorter anterior flagellum is attached to a pronounced proboscis that comprises approximately half of the cell length and moves in a sweeping up‐ and down motion. Cell body ovate, somewhat depressed, very slightly changeable in form, about twice as long as broad. Contractile vesicle apparently single, situated near the anterior extremity (Stokes 1888). Individual species within the six distinct clades are morphologically not clearly distinguishable from each other.

**Type species**. *R. nasuta* (Stokes 1888) Klebs 1892 emend. Wagenhofer, Nitsche & Arndt.

#### 6.1.1 *Rhynchomonas nasuta* (Stokes 1888) Klebs 1892 emend. Wagenhofer, Nitsche & Arndt

**Diagnosis**. “Body ovate, smooth, somewhat depressed, very slightly changeable in form, about twice as long as broad, the posterior extremity, rounded, the anterior produced into a stout, undulating flagellum less than one‐half as long as the zooid; posterior or trailing flagellum slender, about twice as long as the body, arising from the ventral surface at some distance from the frontal border; contractile vesicle apparently single, situated near the anterior extremity; nucleus presumably subcentrally located. Length of body 5–6 μm. Habitat standing pond water” (Stokes 1888). Cells of the neotype are about 3.6–5.8 μm long and about 3.1–4.3 μm wide, the long posterior flagellum measures 9.7–17.1 μm in length. The nucleus measures 1.4 ± 0.3 μm^2^. The ratio of cell area (μm^2^) to posterior flagellum length (μm) is 1.1 ± 0.1. Salinity tolerances 0–11 PSU.

**Neotype location**. Ecological Research Station on the river Rhine at Cologne, Germany (50°54′25.218 N, 6°58′42.1392 E). Benthic habitat, riverine freshwater.

**Neotype material**. The type culture is currently deposited in the Heterotrophic Flagellate Collection Cologne, as isolate HFCC2102, specimen shown in Figure 7A. SEM filters are deposited at the Biology Centre of the Museum of Natural History in Upper Austria, Linz, at number OLML 2026/11.

**Gene sequence data**. The 18S rDNA sequence has been deposited at GenBank with the Accession Number PZ437283.

**ZooBank ID**. LSID urn:lsid:zoobank.org:act:133BABF1‐E9A8‐482E‐ADB6‐09BEB24F23D5.

#### 6.1.2 *Rhynchomonas coloniensis* n. sp. Wagenhofer, Nitsche & Arndt

**Diagnosis**. Cells of this species are typically ovoid, ventrally flattened, 4.3–6.4 μm long, 2.6–3.8 μm wide (during forward motion) and move at a slow pace. The long posterior flagellum measures 11.3–17.1 μm in length and the nucleus measures 1.2 ± 0.3 μm^2^. The ratio of cell area (μm^2^) to posterior flagellum length (μm) is 0.9 ± 0.1. Salinity tolerance ranges between 0 and 5 PSU.

**Remarks**. All isolates belonging to *Rhynchomonas coloniensis* n. sp. were isolated from the river Rhine at Cologne, Germany. P‐distances (SSU rDNA) of type isolate HFCC2101 to the next closest isolates were 1.90% (*Rhynchomonas nasuta*, AY490214), 2.72% (*Rhynchomonas* sp., AY425023), and 3.12% (*Rhynchomonas* sp., AY998642). P‐distances (SSU rDNA) to rhynchomonad isolates from other clades exceed 4.51% (up to 7.63%).

**Type locality**. Ecological Research Station on the river Rhine at Cologne, Germany (50°54′25.218 N, 6°58′42.1392 E). Benthic habitat, riverine freshwater.

**Type material**. The type culture is currently deposited in the Heterotrophic Flagellate Collection Cologne, as isolate HFCC2101, specimen shown in Figure 7G. Electron microscopy preparation of HFCC2101 has been deposited in the Biology Centre of the Museum of Natural History in Upper Austria, Linz, at number OLML 2026/12.

**Gene sequence data**. The sequences (SSU and LSU rDNA) of *Rhynchomonas coloniensis* n. sp. isolate HFCC2101 have been deposited in GenBank with the accession numbers PZ437284 (SSU rDNA), PZ481015 (LSU rDNA).

**Etymology**. Species name refers to the origin of the isolate, the city of Cologne, Germany.

**ZooBank ID**. LSID urn:lsid:zoobank.org:act:BD641C16‐722A‐4B93‐8 AD9‐E494D8A39FD1.

#### 6.1.3 *Rhynchomonas minimamirounga* n. sp. Wagenhofer, Nitsche & Arndt

**Diagnosis**. Cells of this species are typically ovoid to elliptical, slightly elongated and ventrally flattened, 3.8–5.6 μm long and 2.7–3.8 μm wide (during forward motion). Movements are often chaotic and nonlinear at medium speed. The long posterior flagellum measures 9.3–15.6 μm in length, and the nucleus measures 0.9 ± 0.2 μm^2^. The ratio of cell area (μm^2^) to posterior flagellum length (μm) is 1.0 ± 0.1. Salinity tolerance ranges between 0.3 and 133 PSU.

**Remarks**. All isolates belonging to *Rhynchomonas minimamirounga* n. sp. were isolated from Atlantic Ocean deep sea samples. HFCC147 from sediment overlying water; the other isolates from sediment samples. P‐distances (SSU rDNA) of type isolate HFCC147 to the next closest species were 2.03% (*Rhynchomonas metabolita*, HFCC111) and 1.54% (*Rhynchomonas reesi* n. sp., HFCC2070). P‐distances (SSU rDNA) to rhynchomonad isolates from other clades exceed 3.75% (up to 7.26%).

**Type locality**. Marine deep‐sediment in the South Atlantic Ocean at 5127 m depth (−14°58′43.2012 N, −29°57′28.1988 E).

**Type material**. The type culture is currently deposited in the Heterotrophic Flagellate Collection Cologne, as isolate HFCC147, specimen shown in Figure 6E. Electron microscopy preparation of HFCC147 has been deposited in the Biology Centre of the Museum of Natural History in Upper Austria, Linz at number OLML 2026/13. Deep‐frozen material is deposited at the Heterotrophic Flagellate Culture Collection Cologne.

**Gene sequence**. The sequences (SSU and LSU rDNA) of *Rhynchomonas minimamirounga* n. sp. isolate HFCC147 have been deposited in GenBank with the accession numbers PZ437281 (SSU rDNA), PZ481012 (LSU rDNA).

**Etymology**. *minima* refers to the Latin term minimus and its declension minima, meaning the smallest. *Mirounga* is the Latin genus name for elephant seals, which, like rhynchomonads, possess a pronounced proboscis.

**ZooBank ID**. LSID urn:lsid:zoobank.org:act:19EE3F46‐AC05‐49BD‐A9B0‐A5CE3145D149.

#### 6.1.4 *Rhynchomonas reesi* n. sp. Wagenhofer, Nitsche & Arndt

**Diagnosis**. Cells of this species have typically an ovoid to rounded shape, are slightly ventrally flattened, 4.1–6.8 μm long and 3.0–5.1 μm wide (during forward motion). Movements are fast, often chaotic, frequently staying attached at same position using the posterior flagellum as anker. The long posterior flagellum measures 10.8–17.7 μm in length and the nucleus measures 1.9 ± 0.4 μm^2^. The ratio of cell area (μm^2^) to posterior flagellum length (μm) is 1.2 ± 0.2. Salinity tolerance ranges between 0 and 35 PSU.

**Remarks**. The two isolates belonging to *Rhynchomonas reesi* n. sp. were isolated from freshwater samples in the Lower Rhine, close to Rees. One isolate is from surface water and the other from a surface sediment sample. P‐distances (SSU rDNA) of type isolate HFCC2058 to the next closest species from Clades I and II were 1.67% (*Rhynchomonas metabolita*, HFCC111) and 1.54% (*Rhynchomonas minimamirounga* n. sp., HFCC147). P‐distances (SSU rDNA) to rhynchomonad isolates from other clades exceed 3.71% (up to 7.65%).

**Type locality**. Surface water (freshwater) of an nutrient‐rich oxbow of the River Rhine, Bienener Altrhein, near Rees (51°48′21.1 N, 6°21′45.4 E).

**Type material**. The type culture is currently deposited in the Heterotrophic Flagellate Collection Cologne, as isolate HFCC2058, specimen shown in Figure 6K. Electron microscopy preparation of HFCC2058 has been deposited in the Biology Centre of the Museum of Natural History in Upper Austria, Linz, at number OLML 2026/14.

**Etymology**. *reesi* refers to the town Rees, where the Ecological Research Station of the Institute of Zoology (University of Cologne) is situated. The isolate was isolated nearby.

**Gene sequence data**. The sequences (SSU and LSU rDNA) of *Rhynchomonas minimamirounga* n. sp. isolate HFCC2058 have been deposited in GenBank with the accession numbers PZ437279 (SSU rDNA), PZ481010 (LSU rDNA).

**ZooBank ID**. LSID urn:lsid:zoobank.org:act:3E62CE47‐7B1B‐42EC‐B724‐125106EF16DB.

#### 6.1.5 *Rhynchomonas metabolita* (Pshenin 1964) emend. Wagenhofer, Nitsche & Arndt

**Basionym: *Rhynchomonas metabolita* Pshenin** 1964

**Diagnosis**. “A marine benthic organism. Grows well in culture together with *Coelosphaerium benticum* in a liquid Fedorov medium for aerobic nitrogen fixers. In culture conditions, it reproduces in large quantities. It differs sharply from planktonic marine species of *Rhynchomonas* in its small size and morphology. In size, morphology and some other features, it is very similar to freshwater and marine forms of *Rh. nasuta* (Stokes) Klebs. However, it differs greatly from them in its clearly expressed body metabolism and its strong compression from the sides. From the side, the body has an ovoid outline and sizes of 3.5–7 μm × 2–4.5 μm (without the proboscis), and from the side of the ‘abdomen’ or ‘back’ it looks like a biconvex lens with a thickness of 1.0–2 μm. The movement of the proboscis is very lively, and the movement of the rhynchomonad from place to place is very fast. The dragging whip can be glued to the substrate with part of its final length. The length of the whip is from 7 to 10 μm. It is not possible to see where the whip starts near the proboscis. It prefers to feed on small (from 0.5 to 1 μm in diameter) cells of *Coelosphaerium benticum*, which are clearly visible in the body of the flagellate. Less often it feeds on bacteria. Swallowing is done with the help of movements of the proboscis. When swallowing and when defecating, the body moves faster and shows metabolic movement. It is found in the mud of the Mediterranean Sea at a depth of 1100–2400 m.” (Pshenin 1964; translated from Russian). Pshenin (1964) also observed the formation of cysts in *Rhynchomonas metabolita* specimens. Cells of isolate HFCC111 resemble the original description. They are typically elongated and elliptical to D‐shaped, slightly ventrally flattened, 4.0–6.1 μm long and 2.8–4.1 μm wide (during forward motion). Forward locomotion is rapid and mostly steady and continuous. The long posterior flagellum measures 11.7–19.2 μm in length (measured from the flagellar pocket) and the nucleus measures 1.1 ± 0.2 μm^2^. The ratio of cell area (μm^2^) to posterior flagellum length (μm) is 0.8 ± 0.1. Salinity tolerance ranges between 0 and 110 PSU.

**Remarks**. The original specimen of *Rhynchomonas metabolita* described by Pshenin (1964) was isolated from the Mediterranean Sea from 1000 to 2000 m depth. P‐distances (SSU rDNA) of type isolate HFCC111 to the next closest species were 1.67% (*Rhynchomonas reesi* n. sp., HFCC2058) and 1.98% (*Rhynchomonas minimamirounga* n. sp., HFCC147). P‐distances (SSU rDNA) from isolates of this species to rhynchomonad isolates from other clades exceed 4.42% (up to 7.06%).

**Neotype location**. Marine deep‐sea hard substrate from the Eastern Mediterranean, Pliny trench (4390 m, 34°25′4.98 N, 26°7′6.1788 E).

**Neotype material**. The neotype culture is currently deposited in the Heterotrophic Flagellate Collection Cologne, as isolate HFCC111, specimen shown in Figure 6B. Electron microscopy preparation of HFCC111 has been deposited in the Biology Centre of the Museum of Natural History in Upper Austria, Linz, at number OLML 2026/15. Deep‐frozen material is deposited at the Heterotrophic Flagellate Culture Collection Cologne.

**Gene sequence data**. The sequence (SSU rDNA) of *Rhynchomonas metabolita* isolate HFCC111 has been deposited in GenBank with the accession number PZ437268 (SSU rDNA).

**Etymology**. *metabolita* refers to the metabolic and shape‐changing capabilities of rhynchomonads (Pshenin 1964).

**ZooBank ID**. urn:lsid:zoobank.org:act:888BD81C‐BD9D‐4DE0‐9393‐6D6CF60C4FB3.

#### 6.1.6 *Rhynchomonas eder* n. sp. Wagenhofer, Nitsche & Arndt

**Diagnosis**. Cells of this species typically have an elongated elliptical shape, are slightly ventrally flattened, 3.5–6.6 μm long, 2.3–4.0 μm wide (during forward motion) and move fast and straight‐lined. The long posterior flagellum measures 11.6–18.2 μm in length and the nucleus measures 1.3 ± 0.3 μm^2^. The ratio of cell area (μm^2^) to posterior flagellum length (μm) is 0.9 ± 0.1. Salinity tolerance ranges between 15 and 75 PSU.

**Remarks**. The three isolates belonging to *Rhynchomonas eder* n. sp. were isolated from marine (2) or brackish (1) sediment samples. P‐distances (SSU rDNA) of type isolate HFCC1570 to the closest clustering isolate were 3.45% (*Rhynchomonas* sp., HFCC656). P‐distances (SSU rDNA) to rhynchomonad isolates from other clades exceed 3.15% (up to 7.80%).

**Type material**. The type culture is currently deposited in the Heterotrophic Flagellate Collection Cologne, as isolate HFCC1570, specimen shown in Figure 7M. Electron microscopy preparation of HFCC1570 has been deposited in the Biology Centre of the Museum of Natural History in Upper Austria, Linz, at number OLML 2026/16.

**Type locality**. Marine deep‐sea sediment (997 m) in the North Atlantic Ocean off Porto Santo, Madeira Archipelago (33°0′22 N, −16°20′50 E).

**Gene sequence data**. The sequences (SSU and LSU rDNA) of *Rhynchomonas eder* n. sp. isolate HFCC1570 have been deposited in GenBank with the accession numbers PZ437288 (SSU rDNA), PZ481017 (LSU rDNA).

**Etymology**. *eder* relates to the widespread South German name Eder that was the topographical name for persons colonizing and inhabiting uncultivated land. This resembles the capacity of rhynchomonads as early colonizers of substrates and biofilms.

**ZooBank ID**. LSID urn:lsid:zoobank.org:act:10E50E7F‐01EB‐4FEE‐B1A2‐6FEA9DBD174F.

#### 6.1.7 *Rhynchomonas mylnikovi* n. sp. Wagenhofer, Nitsche & Arndt

**Diagnosis**. Cells of this species are typically ovoid or egg‐shaped, ventrally flattened, 4.1–6.2 μm long and 2.8–4.5 μm wide (during forward motion). Forward locomotion is rapid and often hectic and chaotic. The long posterior flagellum measures 13.5–24.2 μm in length and the nucleus measures 1.8 ± 0.4 μm^2^. The ratio of cell area (μm^2^) to posterior flagellum length (μm) is 0.9 ± 0.1. Salinity tolerance ranges between 0 and 19 PSU.

**Remarks**. P‐distances (SSU rDNA) of type isolate HFCC17 to the next closest species were 0.99% (*Rhynchomonas iris* n. sp., HFCC1139), 1.38% (*Rhynchomonas baltica* n. sp., HFCC1657), and 1.20% (*Rhynchomonas pyschnyi* n. sp., HFCC1435). P‐distances (SSU rDNA) to rhynchomonad isolates from other clades exceed 4.39% (up to 7.65%).

**Type locality**. Dystrophic freshwater pond in Borok Yaroslavl, Russia (58°3′42.99984 N, 38°14′4.99992 E).

**Type material**. The type culture is currently deposited in the Heterotrophic Flagellate Collection Cologne, as isolate HFCC17, specimen shown in Figure 8C. Electron microscopy preparation of HFCC17 has been deposited in the Biology Centre of the Museum of Natural History in Upper Austria, Linz, at number OLML 2026/17. Deep‐frozen material is deposited at the Heterotrophic Flagellate Culture Collection Cologne.

**Gene sequence data**. The sequences (SSU and LSU rDNA) of *Rhynchomonas mylnikovi* n. sp. isolate HFCC17 have been deposited in GenBank with the accession numbers DQ207595 (SSU rDNA), PZ481025 (LSU rDNA).

**Etymology**. Species is named after the Alexander Mylnikov (1952–2019) in honor of his major contributions to the field of protistology. He originally collected isolate HFCC17.

**ZooBank ID**. LSID urn:lsid:zoobank.org:act:5035520A‐6A13‐419F‐B6E5‐A9A6C084C952.

#### 6.1.8 *Rhynchomonas baltica* n. sp. Wagenhofer, Nitsche & Arndt

**Diagnosis**. Cells of this species have typically an ovoid to rounded shape, are slightly ventrally flattened, 4.3–6.7 μm long and 3.4–5.5 μm wide (during forward motion). Movements are carried out at medium speed and are often hectic and chaotic. The long posterior flagellum measures 12.2–18.9 μm in length and the nucleus measures 1.7 ± 0.5 μm^2^. The ratio of cell area (μm^2^) to posterior flagellum length (μm) is 1.2 ± 0.2. Salinity tolerance ranges between 0 and 75 PSU.

**Remarks**. All three isolates belonging to *Rhynchomonas baltica* n. sp. were isolated from the brackish Baltic Sea sediments. P‐distances (SSU rDNA) of type isolate HFCC1657 to the next closest species were 1.38% (*Rhynchomonas mylnikovi* n. sp., HFCC17), 1.83% (*Rhynchomonas pyschnyi* n. sp., HFCC1435), and 1.48% (*Rhynchomonas iris* n. sp., HFCC1139). P‐distances (SSU rDNA) to rhynchomonad isolates from other clades exceed 3.97% (up to 7.92%).

**Type locality**. Brackish water (12 PSU) sediment of Rønnebank, Baltic Sea (54°46′23.394 N, 14°0′55.6992 E).

**Type material**. The type culture is currently deposited in the Heterotrophic Flagellate Collection Cologne, as isolate HFCC1657, specimen shown in Figure 8K. Electron microscopy preparation of HFCC1657 has been deposited in the Biology Centre of the Museum of Natural History in Upper Austria, Linz, at number OLML 2026/18. Deep‐frozen material is deposited at the Heterotrophic Flagellate Culture Collection Cologne.

**Gene sequence data**. The sequences (SSU and LSU rDNA) of *Rhynchomonas baltica* n. sp. isolate HFCC1657 have been deposited in GenBank with the accession numbers PZ437296 (SSU rDNA), PZ481022 (LSU rDNA).

**Etymology**. *baltica* refers to the origin of the isolate, the Baltic Sea.

**ZooBank ID**. LSID urn:lsid:zoobank.org:act:91347E7F‐DBFB‐48AF‐9130‐F45735EC5FDA.

#### 6.1.9 *Rhynchomonas iris* n. sp. Wagenhofer, Nitsche & Arndt

**Diagnosis**. Cells of this species are ovoid, egg‐shaped to roundish, slightly ventrally flattened, 4.5–6.5 μm long, 3.3–4.9 μm wide (during forward motion), and move at medium pace performing chaotic and hectic movements. The long posterior flagellum measures 8.7–16.1 μm in length, and the nucleus measures 1.4 ± 0.5 μm^2^. The ratio of cell area (μm^2^) to posterior flagellum length (μm) is 1.4 ± 0.2. Salinity tolerance ranges between 0 and 82 PSU.

**Remarks**. Isolates belonging to *Rhynchomonas iris* n. sp. were isolated from Atlantic seawater samples. P‐distances (SSU rDNA) of type isolate HFCC1139 to the next closest isolates were 1.32% (*Rhynchomonas* sp., AY425025), 1.48% (*Rhynchomonas baltica* n. sp., HFCC1657), 1.34% (*Rhynchomonas pyschnyi* n. g. et n. sp., HFCC1435), 0.99% (*Rhynchomonas mylnikovi* n. sp., HFCC17), and 1.53% (*Rhynchomonas* sp., HFCC1659). P‐distances (SSU rDNA) to rhynchomonad isolates from other clades exceed 4.24% (up to 7.46%).

**Type locality**. Deep‐sediment North Atlantic Ocean (2500 m, 18°10′0.06 N, −51°0′3.9 E).

**Type material**. The type culture is currently deposited in the Heterotrophic Flagellate Collection Cologne, as isolate HFCC1139, specimen shown in Figure 9B. Electron microscopy preparation of HFCC1139 has been deposited in the Biology Centre of the Museum of Natural History in Upper Austria, Linz, at number OLML 2026/19.

**Gene sequence data**. The sequences (SSU and LSU rDNA) of *Rhynchomonas iris* n. sp. isolate HFCC1139 have been deposited in GenBank with the accession numbers PZ437295 (SSU rDNA), PZ481021 (LSU rDNA).

**Etymology**. Type isolate HFCC1139 was taken onboard the RV Maria S. Merian. During a second cruise within the same project framework, a crew member named Iris Seidel passed away in a tragic accident. Naming the species after her shall help to remember her warm and supportive personality.

**ZooBank ID**. LSID urn:lsid:zoobank.org:act:A222E92D‐2B2E‐4633‐831C‐7D8C220DAA82.

#### 6.1.10 *Rhynchomonas pyschnyi* n. sp. Wagenhofer, Nitsche & Arndt

**Diagnosis**. Cells of this species are typically elliptical to D‐shaped, slightly ventrally flattened, 3.4–6.8 μm long and 2.3–4.0 μm wide (during forward motion). Movements are often fast and hectic. The long posterior flagellum measures 8.8–15.8 μm in length and the nucleus measures 1.4 ± 0.5 μm^2^. The ratio of cell area (μm^2^) to posterior flagellum length (μm) is 1.1 ± 0.2. Salinity tolerance ranges between 0 and 75 PSU.

**Remarks**. The only isolate belonging to *Rhynchomonas pyschnyi* n. sp. was isolated from the brackish Baltic Sea. P‐distances (SSU rDNA) of type isolate HFCC1435 to the next closest species were 1.17% (*Rhynchomonas* sp., HFCC1347), 1.83% (*Rhynchomonas baltica* n. sp., HFCC1657), 1.34% (*Rhynchomonas iris* n. sp., HFCC1139), 1.20% (*Rhynchomonas mylnikovi* n. sp., HFCC17), and 1.10% (*Rhynchomonas* sp., HFCC752). P‐distances (SSU rDNA) to rhynchomonad isolates from other clades exceed 4.61% (up to 7.77%).

**Type locality**. Brackish water (8 PSU) sediment from Oderbank, Baltic Sea (54°15′0.1332 N, 14°20′57.3468 E).

**Type material**. The type culture is currently deposited in the Heterotrophic Flagellate Collection Cologne, as isolate HFCC1435, specimen shown in Figure 9G. Electron microscopy preparation of HFCC1435 has been deposited in the Biology Centre of the Museum of Natural History in Upper Austria, Linz, at number OLML 2026/20. Deep‐frozen material is deposited at the Heterotrophic Flagellate Culture Collection Cologne.

**Gene sequence data**. The sequences (SSU and LSU rDNA) of *Rhynchomonas pyschnyi* n. sp. isolate HFCC1435 have been deposited in GenBank with the accession numbers PZ437291 (SSU rDNA), PZ481019 (LSU rDNA).

**Etymology**. Species is dedicated to Anke Pyschny, in honor of her permanent outstanding technical support for the protozoological studies and her kindness.

**ZooBank ID**. LSID urn:lsid:zoobank.org:act:E7F04CD7‐750E‐4550‐AB22‐36905E473378.

#### 6.1.11 *Rhynchomonas oltome* n. sp. Wagenhofer, Nitsche & Arndt

**Diagnosis**. Cells of this species have typically an ovoid or egg shape, often also appearing rounded, are ventrally flattened, 5.0–7.1 μm long and 3.4–4.6 μm wide (during forward motion). Movements are often slow, frequently staying attached at the same position using the posterior flagellum as anchor, especially when exposed to temperatures around 20°C. In contrast to most other rhynchomonad isolates, cells seem to prefer colder temperatures (10°C) and do not cope well with higher temperatures for extended periods. The long posterior flagellum measures 11.8–18.9 μm in length and the nucleus measures 2.3 ± 0.5 μm^2^. The ratio of cell area (μm^2^) to posterior flagellum length (μm) is 1.3 ± 0.1. Salinity tolerance ranges between 2.5 and 55 PSU.

**Remarks**. P‐distances (SSU rDNA) of type isolate HFCC99 to the next closest isolates were 4.53% (*Rhynchomonas* sp., HFCC1143) and 4.45% (*Rhynchomonas* sp., KX420740). P‐distances (SSU rDNA) to rhynchomonad isolates from other clades exceed 6.33%.

**Type locality**. Marine deep‐sea sediment from the North Atlantic Ocean (2484 m, 79°4′15.6 N, 4°9′7.2 E).

**Type material**. The type culture is currently deposited in the Heterotrophic Flagellate Collection Cologne, as isolate HFCC99, specimen shown in Figure 9N. Electron microscopy preparation of HFCC99 has been deposited in the Biology Centre of the Museum of Natural History in Upper Austria, Linz, at number OLML 2026/21.

**Gene sequence data**. The sequences (SSU and LSU rDNA) of *Rhynchomonas oltome* n. sp. isolate HFCC99 have been deposited in GenBank with the accession numbers AY827855 (SSU rDNA), PZ481026 (LSU rDNA).

**Etymology**. *oltome* refers to the Maasai term for the African Elephant (oltome). Both organisms, though completely different in size, share the similarity of a pronounced proboscis.

**ZooBank ID**. LSID urn:lsid:zoobank.org:act:A25189B0‐CE32‐475A‐82F3‐2B03F9A70962.

## Author Contributions

**Julian Wagenhofer:** data curation, formal analysis, investigation, visualization, writing – original draft. **Frank Nitsche:** investigation, supervision, writing – review and editing. **Hartmut Arndt:** conceptualization, investigation, supervision, funding acquisition, project administration, writing – review and editing.

## Funding

This work was supported by Deutsche Forschungsgemeinschaft (AR288/5, AR288/10, AR288/16, AR288/23, MerMet 17‐11, 268236062; SFB 1211; B03, B02, NI1097/4), Bundesministerium für Forschung, Technologie und Raumfahrt (03G0828A, 03G0237B, 03F0848A, 03F0937D), and Heinrich Böll Stiftung.

## Supporting information


**Figure S1:** Cutout of the SSU rDNA alignment showing the position and length of the long intron in the 18S of about 550 nucleotides length, for each of the six clades. Nucleotide position is indicated on the top. Clades III and IV do not include any isolates with an intron at this position. There are 33 isolates with an intron in Clade I, four isolates in Clade II, and one isolate each for Clades V and VI. The different colors indicate the four different nucleotides: C (blue), G (black), A (green), T (red).
**Figure S2:** Phylogenetic tree of rhynchomonads based on untrimmed stand‐alone SSU rDNA alignment. Bootstrap values from maximum likelihood analysis (1000 replicates) are depicted at the nodes.
**Figure S3:** Phylogenetic tree of rhynchomonads based on trimmed (Gblocks) stand‐alone SSU rDNA alignment. Bootstrap values from maximum likelihood analysis (1000 replicates) are depicted at the nodes.
**Figure S4:** Salinity preference of each isolate. Freshwater isolates are surrounded by the green box, brackish isolates by the light blue box, and marine isolates by the dark blue box. Experiments were carried out in triplicates and evaluated qualitatively (1 dead–5 highly viable and reproducing). Controls are indicated by the red dots. Treatments are indicated by the black dots. The black dots indicate the average quality score. If the viability among the triplicates differed, the minimum and maximum scores are given as range indicators. Each “X” indicates that no more viable cells were observed in the treatment. A dashed line indicates that only 2/3 treatments were still alive. A dotted line indicates that only 1/3 treatments was still alive.
**Figure S5:** Georeferenced records of *Rhynchomonas* morphotype (GBIF Secretariat 2023).
**Table S1:** Measurements of morphological parameters (mean values ± SD) of all investigated isolates.
**Table S2:** P‐distances (SSU rDNA) of Rhynchomonadidae Clade I isolates calculated with the pairwise alignment function from BioEdit. Identifiers refer to internal HFCC database or GenBank accession numbers.
**Table S3:** P‐distances (SSU rDNA) of Rhynchomonadidae Clade II isolates calculated with the pairwise alignment function from BioEdit. Identifiers refer to internal HFCC database or GenBank accession numbers.
**Table S4:** P‐distances (SSU rDNA) of Rhynchomonadidae Clade III isolates calculated with the pairwise alignment function from BioEdit. Identifiers refer to internal HFCC database or GenBank accession numbers.
**Table S5:** P‐distances (SSU rDNA) of Rhynchomonadidae Clade IV isolates calculated with the pairwise alignment function from BioEdit. Identifiers refer to internal HFCC database or GenBank accession numbers.
**Table S6:** P‐distances (SSU rDNA) of Rhynchomonadidae Clade V isolates calculated with the pairwise alignment function from BioEdit. Identifiers refer to internal HFCC database or GenBank accession numbers.
**Table S7:** P‐distances (SSU rDNA) of Rhynchomonadidae Clade VI isolates calculated with the pairwise alignment function from BioEdit. Identifiers refer to internal HFCC database or GenBank accession numbers.
**Table S8:** P‐distances (18S rDNA and 28S rDNA) of selected Rhynchomonadidae isolates from all six clades calculated with the pairwise alignment function from BioEdit. Identifiers refer to internal HFCC database. The respective alignment on which the p‐distance calculation is based, is given in the Supporting Information. The three values for each comparison refer to the 18S RDNA (top), 28S rDNA (middle), and the combination of both (bottom). SSU p‐distances are based on an approximately 2000n comparison and LSU p‐distances on 650–1100n. An intron of medium‐length (~35n) was removed from the LSU for p‐distance calculations.

**Data S1:** Alignment.

## Data Availability

The data that support the findings of this study are openly available in GenBank at https://www.ncbi.nlm.nih.gov/genbank/about/.
