# Supplementary material for: Taxonomic Revision of the Globally Widespread Cryptic Rhynchomonas Morphospecies Complex
Source: J Eukaryot Microbiol. 2026 Nov 6;73(6):e70137. doi: 10.1111/jeu.70137 (PMC13741200; doi:10.1111/jeu.70137)
Supplement: Supplementary file 1 [file JEU-73-e70137-s002.pdf]

Supplementary Material

Taxonomic revision of the globally widespread cryptic *Rhynchomonas* morphospecies complex

Julian Wagenhofer, Frank Nitsche & Hartmut Arndt

University of Cologne, Institute of Zoology, General Ecology, Zuelpicher Straße 47b, 50674 Cologne, Germany

Supplementary Table 1. Measurements of morphological parameters (mean values +/- S.D.) of all investigated isolates.

HFCC	Clade	Length of cells		Width of cells		Cell area		Length of posterior flagellum		Nucleus area		Ratio of area cell/ posterior flagellum	
		(µm)	n	(µm)	n	(µm²)	n	(µm)	n	(µm²)	n	(µm)	n
18	1	5,990 ± 0,779	20	4,174 ± 0,495	20	19,869 ± 4,675	20	14,128 ± 1,947	20	1,833 ± 0,397	18	1,412 ± 0,302	20
111	1	4,811 ± 0,692	22	3,533 ± 0,370	20	13,426 ± 3,068	20	15,800 ± 2,423	20	1,078 ± 0,203	19	0,844 ± 0,099	20
148	1	5,530 ± 0,585	20	3,385 ± 0,274	20	14,770 ± 2,452	20	13,279 ± 1,649	20	1,409 ± 0,410	11	1,114 ± 0,135	20
1420	1	7,378 ± 0,980	20	4,220 ± 0,558	20	24,638 ± 5,476	20	16,153 ± 2,158	20	2,649 ± 0,623	19	1,513 ± 0,192	20
147	2	4,946 ± 0,589	20	3,268 ± 0,315	20	12,757 ± 2,284	20	12,674 ± 1,811	20	0,923 ± 0,220	13	1,003 ± 0,083	20
2058	2	5,682 ± 0,675	20	4,170 ± 0,532	20	18,823 ± 4,243	20	15,407 ± 1,795	20	1,948 ± 0,432	20	1,211 ± 0,181	20
2101	3	5,309 ± 0,689	20	3,142 ± 0,382	20	13,243 ± 3,072	20	14,372 ± 1,872	20	1,199 ± 0,303	20	0,915 ± 0,130	20
2102	3	5,025 ± 0,623	20	3,772 ± 0,320	20	15,006 ± 2,863	20	14,032 ± 2,201	20	1,389 ± 0,315	20	1,066 ± 0,096	20
2052	3	5,229 ± 0,817	20	3,469 ± 0,408	20	14,392 ± 3,333	20	15,383 ± 2,972	20	1,628 ± 0,369	18	0,931 ± 0,107	20
1570	4	5,420 ± 0,742	20	3,081 ± 0,463	20	13,109 ± 2,697	20	15,353 ± 1,570	20	1,322 ± 0,347	17	0,852 ± 0,146	20
17	5	5,244 ± 0,673	20	3,761 ± 0,461	20	15,648 ± 3,576	20	17,863 ± 3,376	20	1,761 ± 0,392	18	0,876 ± 0,121	20
1139	5	5,475 ± 0,609	20	3,951 ± 0,384	20	17,121 ± 3,359	20	12,273 ± 1,885	20	1,366 ± 0,505	17	1,396 ± 0,179	20
1435	5	5,251 ± 0,874	20	3,421 ± 0,446	20	14,244 ± 3,324	20	12,604 ± 1,869	20	1,360 ± 0,500	12	1,121 ± 0,176	20
1657	5	5,623 ± 0,589	20	4,210 ± 0,502	20	18,763 ± 3,986	20	15,251 ± 1,973	20	1,656 ± 0,464	20	1,229 ± 0,195	20
99	6	5,749 ± 0,554	20	4,025 ± 0,348	20	18,229 ± 2,714	20	14,547 ± 2,106	20	2,270 ± 0,487	18	1,259 ± 0,141	20

Supplementary Table 2. P-distances (SSU rDNA) of Rhynchomonadidae clade I isolates calculated with the pairwise alignment function from BioEdit. Identifiers refer to internal HFCC database or GenBank accession numbers.

	HFCC 830	HFCC 826	HFCC 148	HFCC 1639	HFCC 18	HFCC 111	HFCC 1420	AF174378
HFCC 826	0.39%							
HFCC 148	0.48%	0.66%						
HFCC 1639	0.10%	0.30%	0.59%					
HFCC 18	0.20%	0.37%	0.83%	0.25%				
HFCC 111	0.00%	0.24%	0.48%	0.20%	0.10%			
HFCC 1420	0.00%	0.24%	0.44%	0.15%	0.10%	0.05%		
AF174378	0.77%	0.72%	1.09%	0.54%	0.39%	0.43%	0.39%	
HFCC 1320	0.10%	0.30%	0.58%	0.20%	0.25%	0.19%	0.10%	0.48%

Supplementary Table 3. P-distances (SSU rDNA) of Rhynchomonadidae clade II isolates calculated with the pairwise alignment function from BioEdit. Identifiers refer to internal HFCC database or GenBank accession numbers.

	HFCC 1323	AY425024	HFCC 2058
AY425024	0.97%		
HFCC 2058	0,92%	1.67%	
HFCC 147	1,42%	2.02%	1.04%

Supplementary Table 4. P-distances (SSU rDNA) of Rhynchomonadidae clade III isolates calculated with the pairwise alignment function from BioEdit. Identifiers refer to internal HFCC database or GenBank accession numbers.

	HFCC 2101	HFCC 2102
HFCC 2102	2.01%	
HFCC 234	0.00%	2.01%
AY490214	1.90%	0.55%
AY425023	2.72%	1.76%
AY998642	3.12%	3.15%
HFCC 2052	1.94%	0.20%
HFCC 319	2.00%	0.29%
MW694339	1.92%	0.21%

Supplementary Table 5. P-distances (SSU rDNA) of Rhynchomonadidae clade IV isolates calculated with the pairwise alignment function from BioEdit. Identifiers refer to internal HFCC database or GenBank accession numbers.

	HFCC 656	HFCC 156
HFCC 156	3.36%	
HFCC 1630	3.54%	0.90%

Supplementary Table 6. P-distances (SSU rDNA) of Rhynchomonadidae clade V isolates calculated with the pairwise alignment function from BioEdit. Identifiers refer to internal HFCC database or GenBank accession numbers.

	HFCC 17	HFCC 231	HFCC 1657	AY 425025	HFCC 1139	HFCC 1504	HFCC 1659	HFCC 752	HFCC 1435	HFCC 1347
HFCC 231	0.54%									
HFCC 1657	1.38%	1.45%								
AY425025	1.62%	1.27%	1.43%							
HFCC 1139	0.99%	1.18%	1.48%	1.32%						
HFCC 1504	1.17%	1.27%	1.43%	1.07%	0.20%					
HFCC 1659	1.29%	1.49%	1.93%	1.33%	1.53%	1.58%				
HFCC 752	1.14%	1.40%	1.55%	1.25%	1.15%	1.32%	1.25%			
HFCC 1435	1.20%	1.49%	1.83%	1.43%	1.34%	1.53%	1.14%	1.10%		
HFCC 1347	1.54%	1.47%	1.87%	1.64%	1.69%	1.48%	1.43%	1.30%	1.17%	
HFCC 1658	1.66%	1.63%	1.98%	1.90%	1.64%	1.89%	1.92%	1.98%	2.10%	1.76%

Supplementary Table 7. P-distances (SSU rDNA) of Rhynchomonadidae clade VI isolates calculated with the pairwise alignment function from BioEdit. Identifiers refer to internal HFCC database or GenBank accession numbers.

	HFCC 99	HFCC 1143
HFCC 1143	4.53%	
KX420740	4.45%	2.76%

Supplementary Table 8. P-distances (18S rDNA and 28S rDNA) of selected Rhynchomonadidae isolates from all 6 clades calculated with the pairwise alignment function from BioEdit. Identifiers refer to internal HFCC database. The respective alignment on which the p-distance calculation is based, is given in the supplementary material. The three values for each comparison refer to the 18S RDNA (top), 28S rDNA (middle) and the combination of both (bottom). SSU p-distances are based on an approximately 2000n comparison and LSU p-distances on 650-1100n. An intron of medium-length (~35n) was removed from the LSU for p-distance calculations.

Isolate		HFCC 1307	HFCC 147	HFCC 2070	HFCC 2102	HFCC 2101	HFCC 156	HFCC 1570	HFCC 1657	HFCC 1139
	Clade	1	2	2	3	3	4	4	5	5
HFCC147	2	2.03%<br>1.24%<br>1.79%								
HFCC2070	2	1.58%<br>0.90%<br>1.41%	1.54%<br>1.20%<br>1.46%							
HFCC2102	3	5.03%<br>7.99%<br>5.93%	4.51%<br>8.47%<br>5.67%	4.54%<br>6.98%<br>5.16%						
HFCC2101	3	5.05%<br>8.67%<br>6.16%	4.94%<br>9.00%<br>6.14%	4.69%<br>6.85%<br>5.23%	1.92%<br>2.39%<br>2.06%					
HFCC156	4	4.84%<br>4.30%<br>4.67%	4.02%<br>4.98%<br>4.31%	3.99%<br>4.04%<br>4.00%	3.95%<br>6.43%<br>4.71%	4.53%<br>6.90%<br>5.26%				
HFCC1570	4	4.93%<br>4.80%<br>4.89%	3.91%<br>5.04%<br>4.25%	3.93%<br>4.19%<br>3.99%	3.91%<br>6.64%<br>4.72%	4.51%<br>7.30%<br>5.35%	0.74%<br>0.56%<br>0.69%			
HFCC1657	5	5.56%<br>8.84%<br>6.55%	5.31%<br>8.50%<br>6.27%	4.85%<br>7.92%<br>5.63%	5.02%<br>8.73%<br>6.11%	5.20%<br>9.02%<br>6.33%	3.98%<br>8.06%<br>5.21%	3.97%<br>8.29%<br>5.27%		
HFCC1139	5	5.22%<br>8.46%<br>6.26%	5.05%<br>8.69%<br>6.14%	5.16%<br>7.77%<br>5.82%	4.83%<br>8.00%<br>5.79%	4.77%<br>8.57%<br>5.92%	4.59%<br>9.97%<br>6.47%	4.29%<br>7.92%<br>5.40%	1.48%<br>2.31%<br>1.73%	
HFCC99	6	6.87%<br>10.07%<br>7.89%	6.99%<br>9.92%<br>7.87%	6.95%<br>10.62%<br>7.88%	7.22%<br>11.35%<br>8.47%	6.85%<br>11.65%<br>8.32%	7.19%<br>9.66%<br>7.98%	7.12%<br>9.79%<br>7.93%	6.93%<br>10.33%<br>7.95%	6.33%<br>9.37%<br>7.28%

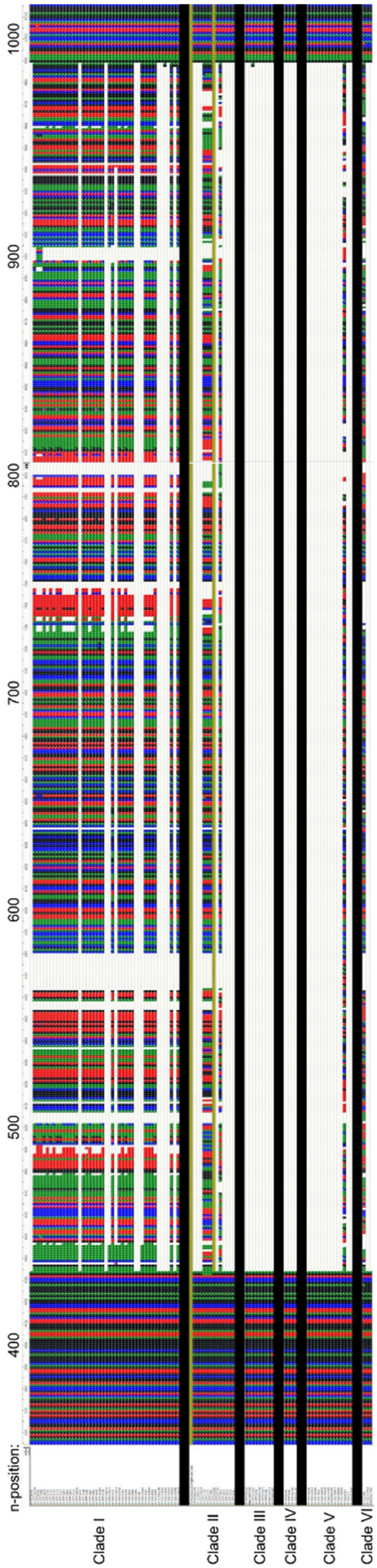

Supplementary Figure 1. Cutout of the SSU rDNA alignment showing the position and length of the long intron in the 18S of about 550 nucleotides length, for each of the six clades. Nucleotide position is indicated on the top. Clades III and IV do not include any isolates with an intron at this position. There are 33 isolates with an intron in clade I, four isolates in clade II and one isolate each for clades V and VI. The different colors indicate the four different nucleotides: C (blue), G (black), A (green), T (red).

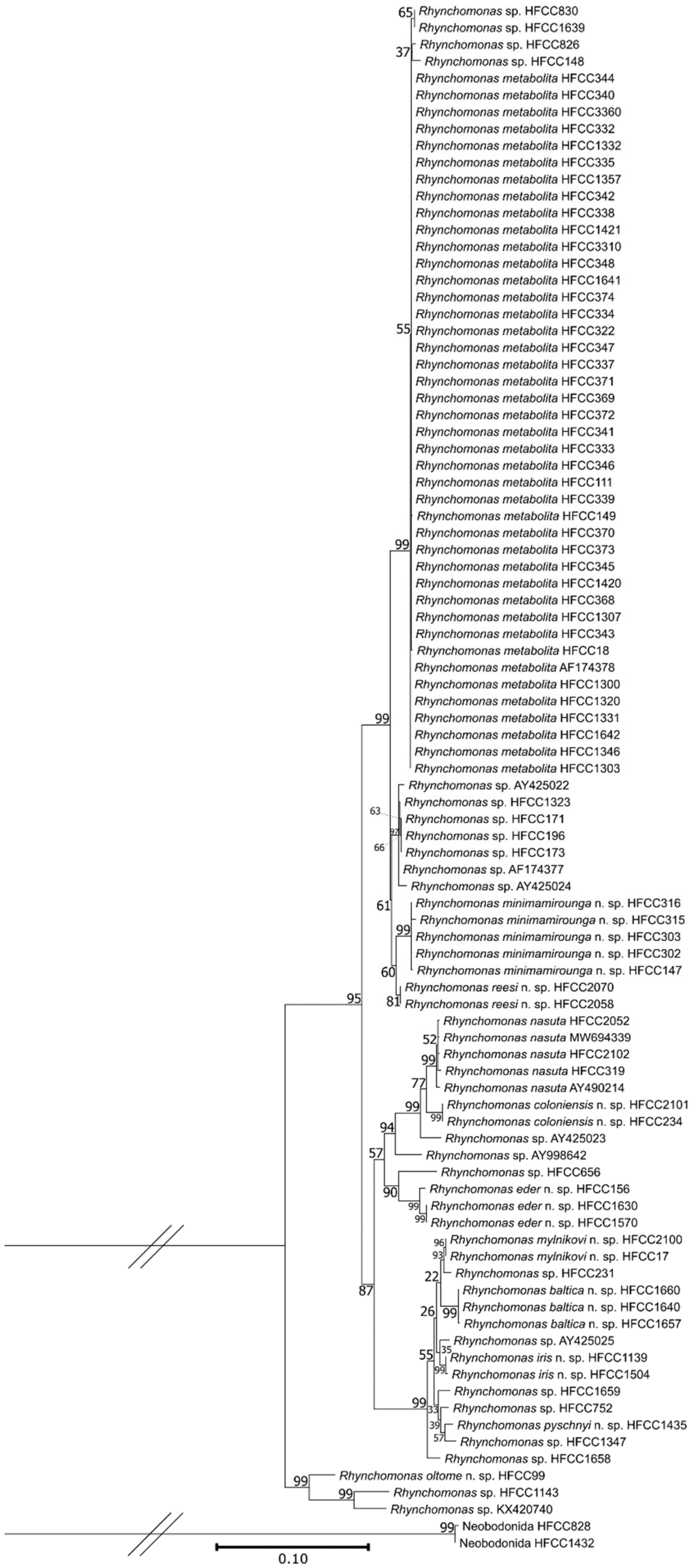

Supplementary Figure 2. Phylogenetic tree of rhynchomonads based on untrimmed stand-alone SSU rDNA alignment. Bootstrap values from Maximum Likelihood analysis (1000 replicates) are depicted at the nodes.

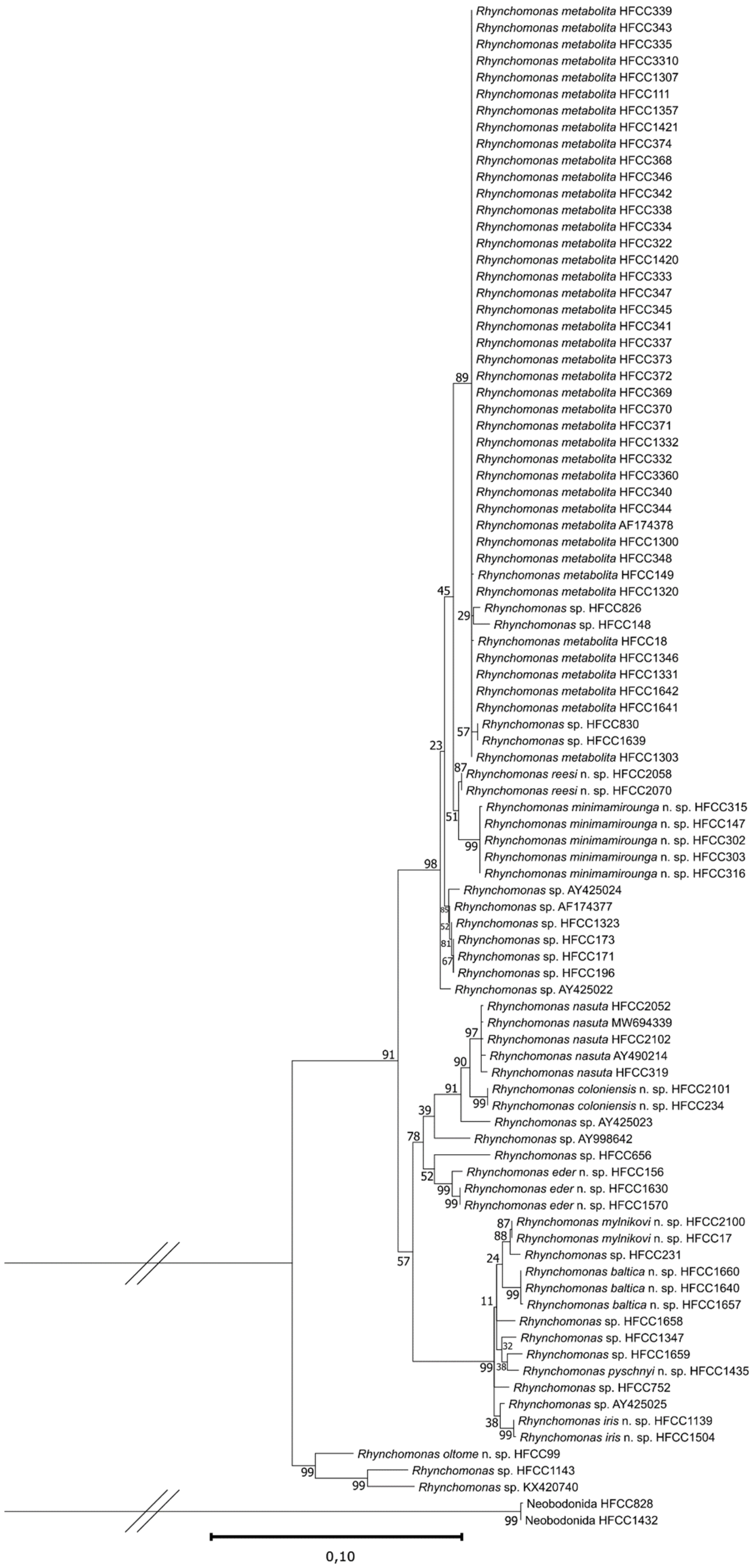

Supplementary Figure 3. Phylogenetic tree of rhynchomonads based on trimmed (Gblocks) stand-alone SSU rDNA alignment. Bootstrap values from Maximum Likelihood analysis (1000 replicates) are depicted at the nodes.

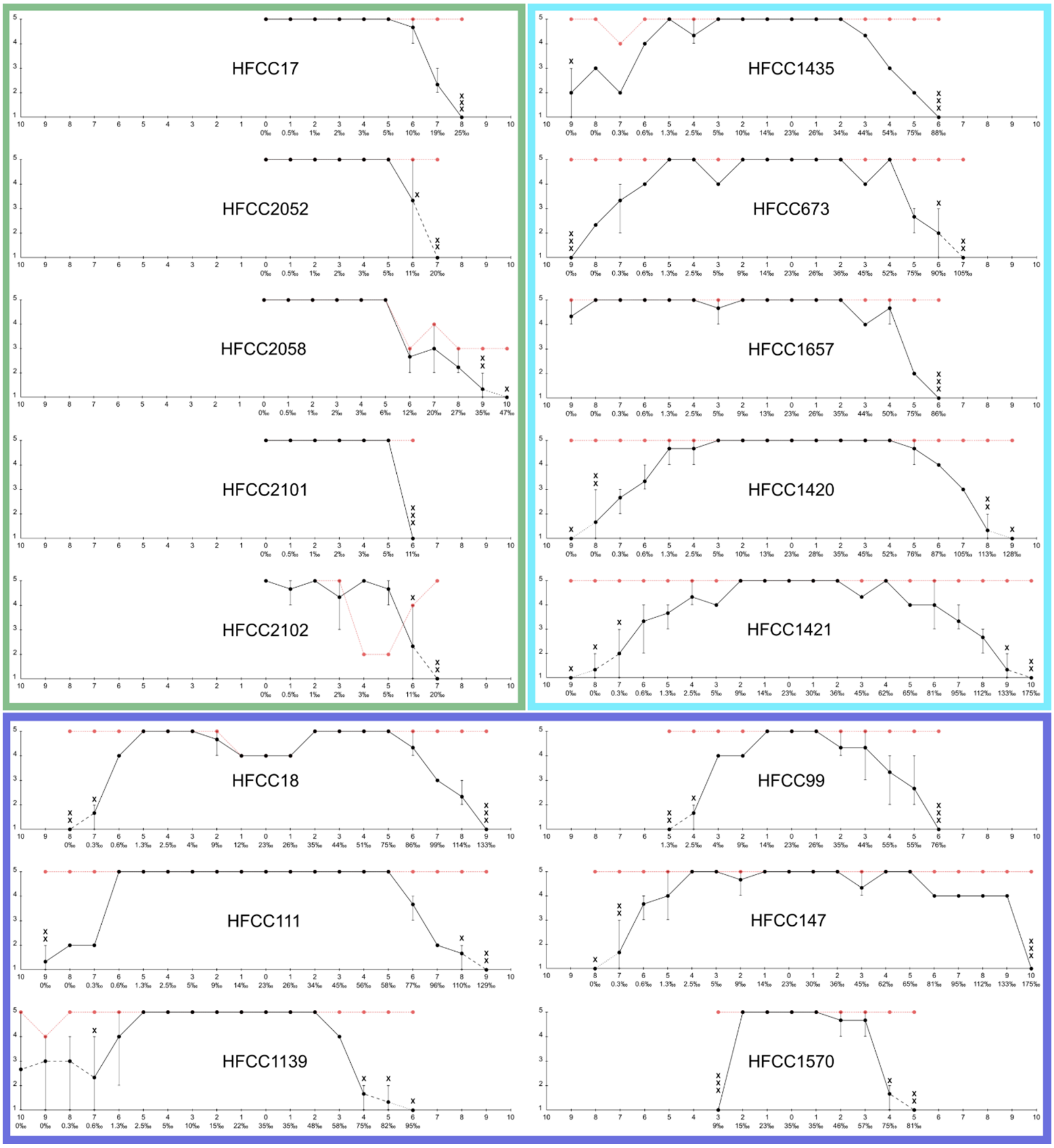

Supplementary Figure 4. Salinity preference of each isolate. Freshwater isolates are surrounded by the green box, brackish isolates by the light blue box and marine isolates by the dark blue box. Experiments were carried out in triplicates and evaluated qualitatively (1 dead – 5 highly viable and reproducing). Controls are indicated by the red dots. Treatments are indicated by the black dots. The black dots indicate the average quality score. If the viability among the triplicates differed, the minimum and maximum scores are given as range indicators. Each "X" indicates that no more viable cells were observed in the treatment. A dashed line indicates that only 2/3 treatments were still alive. A dotted line indicates that only 1/3 treatments was still alive.

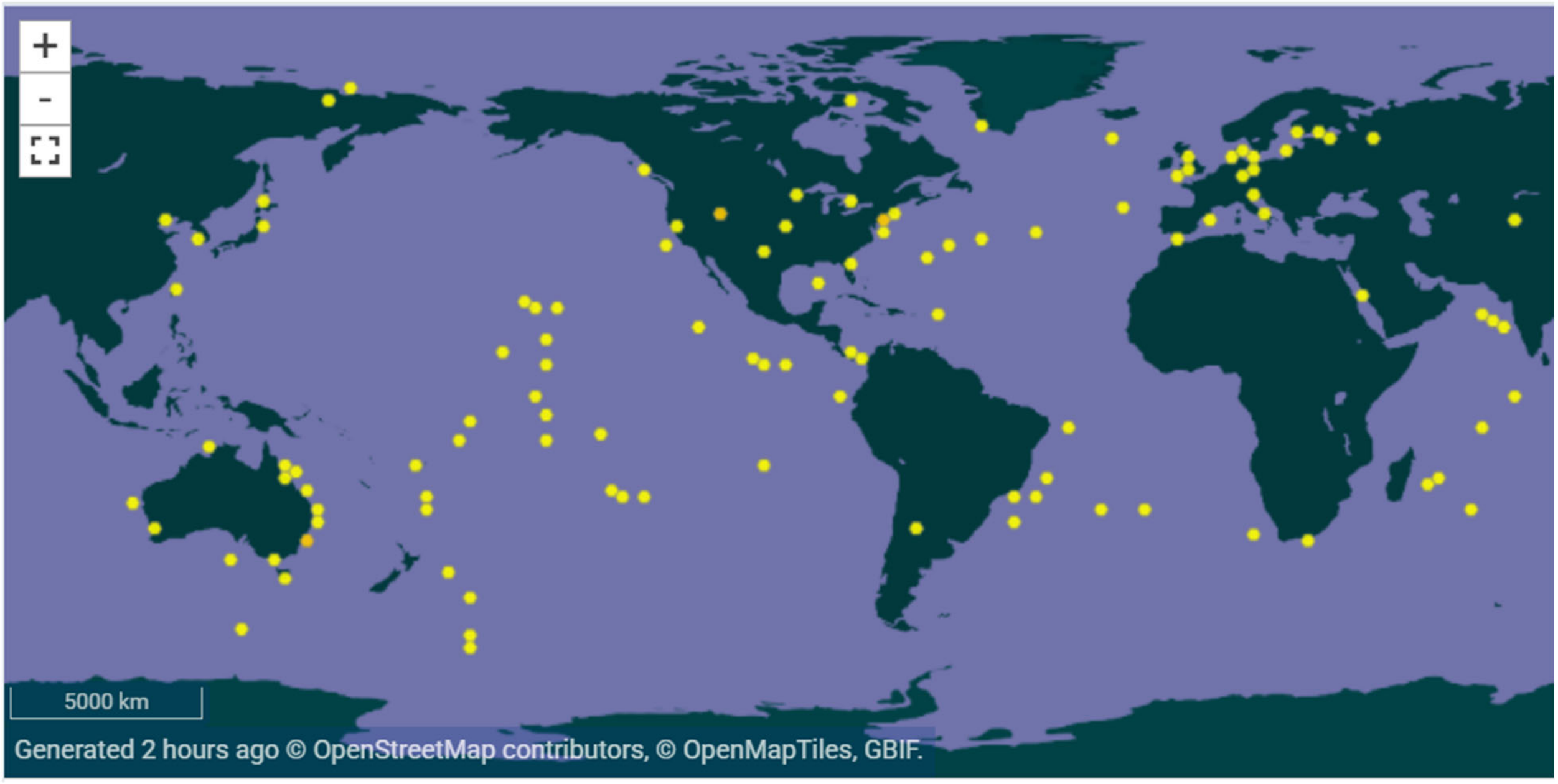

Supplementary Figure 5. Georeferenced records of *Rhynchomonas* morphotype (GBIF Secretariat, 2023).
